# Directed Evolution of Branched-Chain α‑Keto Acid Decarboxylase for 3‑Hydroxypropionic Acid Production in *Escherichia coli* via Oxaloacetate

**DOI:** 10.1021/acssynbio.5c00267

**Published:** 2025-08-15

**Authors:** Chuang Wang, René C. L. Olsthoorn, Huub J. M. de Groot

**Affiliations:** Leiden Institute of Chemistry, 4496 Leiden University, Leiden 2300 RA, The Netherlands

**Keywords:** 3-hydroxypropionate, branched-chain α-keto acid decarboxylase, oxaloacetate pathway, directed evolution, *Escherichia coli*

## Abstract

3-Hydroxypropionic acid (3-HP) serves as a crucial platform chemical with diverse applications across various industries. In this study, the oxaloacetate pathway was utilized for 3-HP production. This pathway involves the decarboxylation of oxaloacetate into malonic semialdehyde, catalyzed by branched-chain α-keto acid decarboxylase (KdcA), which is subsequently reduced to 3-HP by dehydrogenases. Directed evolution of KdcA was carried out to enhance its catalytic efficiency toward oxaloacetate, resulting in a KdcA^M8^ mutant with the following substitutions: S286R, S287T, F381H, F382P, L534S, L535F, M538T, and G539F. Compared to wild-type (WT) KdcA, KdcA^M8^ exhibits a lower *K*_M_ value toward oxaloacetate (*K*_M_ = 1.15 mM vs *K*_M_ > 25 mM). Among these mutations, the single mutants S286R and S287T exhibited 5.5-fold and 1.3-fold increased activities, respectively. Instead of WT KdcA, the KdcA^M8^ mutant was integrated into *Escherichia coli* (*E. coli*) BL21 strain, resulting in the production of 3-HP at a concentration of 0.11 mM. To further improve 3-HP production, two dehydrogenases were compared for the downstream conversion of malonic semialdehyde into 3-HP, and two carboxylases were explored to enhance the upstream precursor supply of oxaloacetate. Additionally, the growth conditions were optimized. Finally, a nonnatural oxaloacetate pathway was successfully engineered in the *E. coli* BL21 strain, achieving a 3-HP titer of approximately 0.71 mM from glucose. This work illustrates that protein engineering is a powerful tool for modulating flux in the target pathway and holds promise for the future development of the oxaloacetate pathway to improve the 3-HP yield.

## Graphical abstract

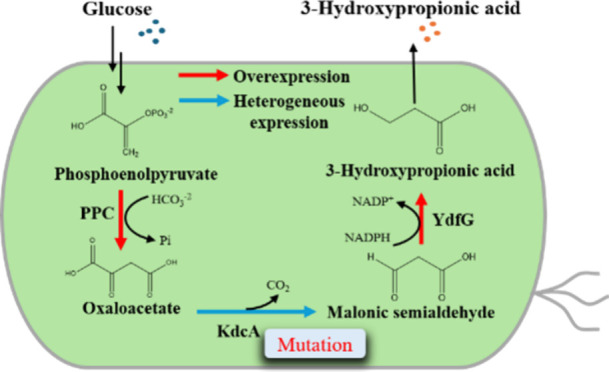

## Introduction

In recent years, there has been a significant focus on producing chemicals, fuels, and materials from renewable resources to achieve economic and social benefits. This shift toward sustainable biomanufacturing is driven by global environmental concerns and an anticipated shortage of fossil resources.^1^ To achieve this, it will be essential to develop efficient biosynthesis methods to enable large-scale production of chemicals.^2^

3-Hydroxypropionic acid (3-HP) is considered a valuable chemical derived from biomass for various industrial applications. It is used as a precursor for the synthesis of several important compounds such as 1,3-propanediol, acrylic acid, malonate, biodegradable polymers, and others.^3,4^ Traditionally, 3-HP has been synthesized through various chemical routes; however, these methods are not commercially feasible due to high costs, the toxicity of raw materials, and environmental concerns.^5,6^ In contrast, biological synthesis of 3-HP using renewable feedstock has attracted significant attention.^4^ Recently, a novel oxaloacetate pathway for 3-HP production was proposed, wherein oxaloacetate is converted to malonic semialdehyde by an α-ketoacid decarboxylase, followed by its reduction to 3-HP.^7^ Two α-ketoacid decarboxylases, benzoylformate decarboxylase (MdlC) from *Pseudomonas putida* (*P. putida*) E23 and branched-chain α-ketoacid decarboxylase (KdcA) from *Lactococcus lactis* (*L. lactis*), have been identified as having activity toward oxaloacetate, with reported activities of 3.420 and 1.825 U/mg, respectively.^7^ MdlC has been successfully applied in the oxaloacetate pathway, achieving the highest reported 3-HP production level of 0.2 M in *Saccharomyces cerevisiae* (*S. cerevisiae*).^7^ However, the potential of KdcA in 3-HP biosynthesis via this pathway has not yet been explored. In the oxaloacetate pathway, α-ketoacid decarboxylase is critical for directing the metabolic flux from oxaloacetate to 3-HP. Both MdlC and KdcA are rate-limiting enzymes in this pathway due to their low activities toward the nonnative substrate, oxaloacetate. Therefore, enhancing the activity and selectivity of either MdlC or KdcA through protein engineering would be beneficial for improving 3-HP production via the oxaloacetate pathway.

Directed evolution, a widely used method for protein engineering, has been very effective in modifying natural enzymes to obtain desired properties.^8,9^ The success of a directed evolution experiment depends on two key factors: the creation of high-quality mutant libraries and the use of efficient high-throughput screening or selection methods. Advances in molecular biology have led to the development of techniques such as random mutagenesis, focused mutagenesis, and gene recombination, enabling the generation of diverse and high-quality mutant libraries.^10^ In parallel, the outcome of directed evolution experiments is critically dependent on the screening or selection methods used to identify the desired mutants.^11^ Therefore, developing effective screening or selection methods is essential.

In a previous study, Liu et al. demonstrated a growth selection system for the directed evolution of the C-terminal domain of malonyl-CoA reductase (MCR-C).^12^ Specifically, a β-alanine auxotrophic strain, BL21­(DE3) Δ*panD*, was generated by deleting the essential gene l-aspartate-α-decarboxylase (*panD*). To restore β-alanine biosynthesis, a synthetic pathway involving MCR-C and β-alanine-pyruvate aminotransferase (BAPAT) as key enzymes was introduced into the host, thereby creating a strong *in vivo* correlation between the host phenotype and MCR-C activity. The results showed that after one round of selection, several active MCR-C variants were isolated, with activity increases ranging from 56 to 92%.^12^ Furthermore, it was suggested that this selection system can automatically eliminate undesired variants, isolating only active variants from the library by monitoring cell growth via cell density in a liquid medium and colony formation or size on a solid medium. Given the efficiency of this selection system, it was proposed that a similar approach could be adopted for the directed evolution of KdcA.

In this study, we explored the biosynthesis of 3-HP via the oxaloacetate pathway in *Escherichia coli* (*E. coli*) (Figure 1A). The KdcA was chosen as the key enzyme in the decarboxylation of oxaloacetate. To improve its catalytic efficiency toward oxaloacetate, the directed evolution of KdcA was employed based on a growth selection system. Initially, both wild-type (WT) KdcA and engineered KdcA were compared in the biosynthesis of 3-HP. To further improve 3-HP production, optimization strategies were implemented. These included comparing the *in vivo* performance of dehydrogenases from *E. coli* and *P. putida* for downstream conversion, overexpressing phosphoenolpyruvate carboxylase and pyruvate carboxylase to increase the supply of the oxaloacetate precursor, and fine-tuning growth conditions.

**Figure 1:**
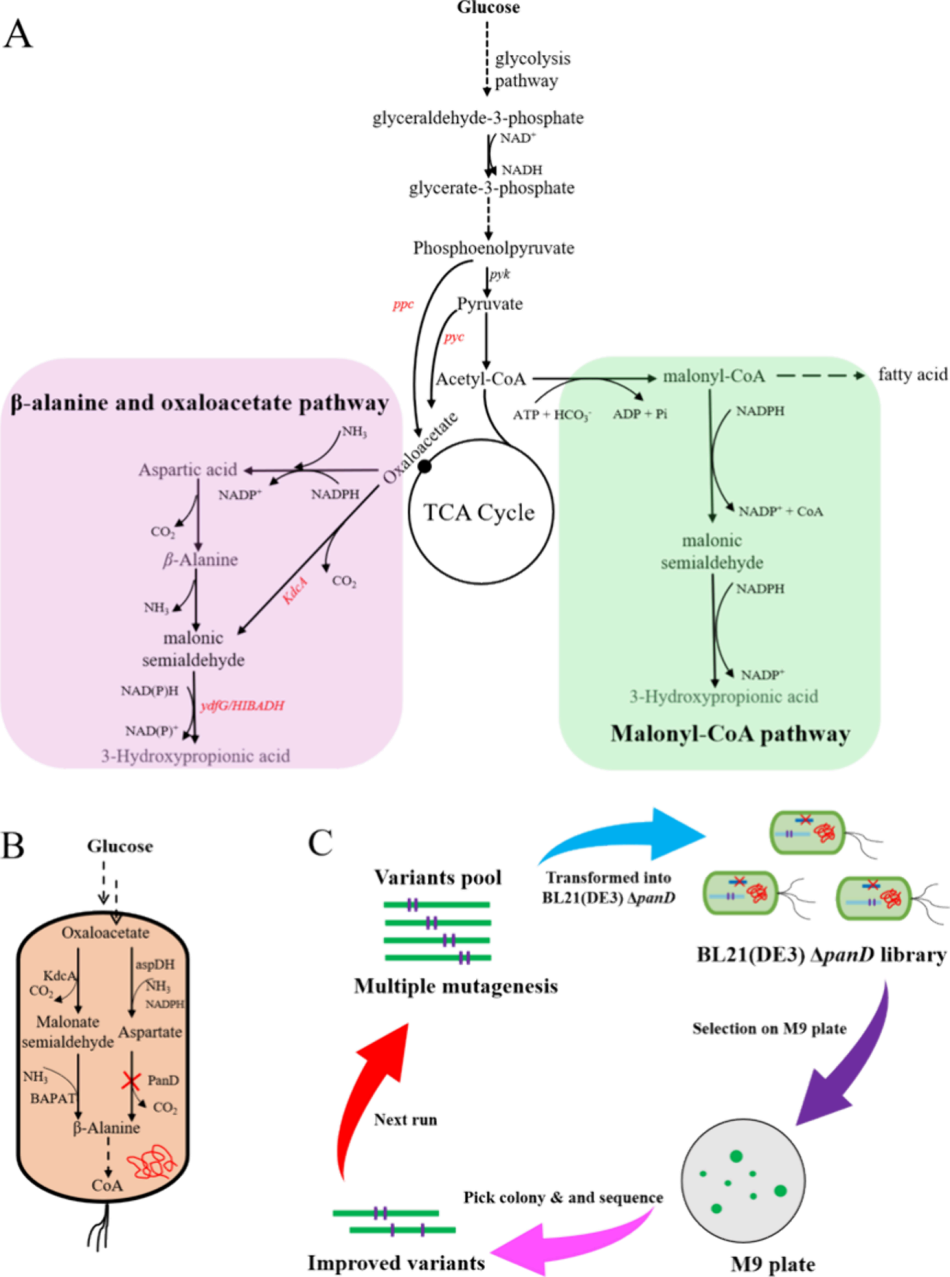
(A) Three main pathways for the biosynthesis of 3-hydroxypropionic acid from glucose. The oxaloacetate pathway in the left panel is used in this study. *ppc*: encoding phosphoenolpyruvate carboxylase, *pyc*: encoding pyruvate carboxylase, *ydfG*: encoding 3-hydroxy acid dehydrogenase, HIBADH: encoding 3-hydroxyisobutyrate dehydrogenase, KdcA: encoding branched-chain α-ketoacid decarboxylase. (B) The growth selection system that was used for the directed evolution of KdcA. β-alanine is expected to be produced from oxaloacetate by introducing exogenous enzymes KdcA and BAPAT, and the resulting β-alanine restores cell growth of the BL21­(DE3) Δ*panD* strain. (C) General procedure for improving the KdcA enzyme through growth-based directed evolution.

## Results and Discussion

### Growth Selection-Based Evolution of KdcA

To achieve the desired catalytic efficiency of KdcA for 3-HP production via the oxaloacetate pathway, a growth selection system was adopted for the directed evolution of KdcA. In this system, the BL21­(DE3) Δ*panD* strain was generated by CRISPR-Cas9-mediated deletion of the *panD* gene, resulting in a strain that is unable to grow in an M9 medium without the addition of β-alanine or pantothenate. Subsequently, an alternative β-alanine biosynthetic pathway was constructed by introducing the genes encoding KdcA and β-alanine-pyruvate aminotransferase (BAPAT) into BL21­(DE3) Δ*panD*. In the engineered BL21­(DE3) Δ*panD* strain, oxaloacetate derived from glucose is converted to malonic semialdehyde by KdcA, which is subsequently converted into alanine by BAPAT (Figure 1B). Under these conditions, cell growth is significantly linked to KdcA, which drives the flux of oxaloacetate into β-alanine. As shown in Supplementary Figure 2, BL21­(DE3) Δ*panD*/pKdcA and BL21­(DE3) Δ*panD*/pBAPAT strains were unable to grow in an M9 medium. Also, the BL21­(DE3) Δ*panD*/pKdcA-BAPAT strain, which coexpresses KdcA and BAPAT, exhibited very limited cell growth in an M9 medium. In contrast, when supplied with 50 μM β-alanine, these strains were able to recover growth in 24 h of incubation. These results suggest that the BL21­(DE3) Δ*panD* strain is potentially a suitable host for the evolution of enzymes (e.g., KdcA) related to the biosynthesis of β-alanine. Furthermore, the inability of the BL21­(DE3) Δ*panD* strain coexpressing KdcA and BAPAT to restore growth indicates that KdcA exhibits low catalytic efficiency toward oxaloacetate.

To increase the chance of success in the directed evolution rounds, we first analyzed the structure of KdcA (PDB: 2VBG) and selected eight residues in the binding pocket (Figure 2A). Among them, residues S286, F381, and M538 reside in the enzyme’s active site and influence catalytic activity.^13^ The neighboring residues S287, F382, and G539 may play similar roles. Additionally, two other residues, L534 and L535, are positioned near the active site and may contribute to substrate recognition and docking. Therefore, mutations at these eight residues were likely to enhance the enzyme’s activity and affinity toward oxaloacetate. These residues were grouped into S286–S287, F381–F382, L534–L535, and M538–G539 for simultaneous saturation mutagenesis, as this pairing strategy facilitated the efficient construction of the mutation library via PCR. Each group was mutated using two NNK codons. The selection process, as outlined in Figure 1C, consisted of four rounds of evolution in BL21­(DE3) Δ*panD*/pKdcA-BAPAT strain, during which positive KdcA variants were identified based on colony formation and size on the solid medium. The first round involved the selection of a mutant library at codons 286–287, with the positive variant selected for the second round of evolution. In the second round, saturation mutagenesis was introduced in the F381–F382 groups. Subsequent rounds of evolution, the third and fourth, focused on introducing mutant libraries at codons 534–535 and 538–539, respectively. Although the mutant libraries were smaller than those created by random mutation or combining different groups, this strategy has been successfully used in directed evolution of amine-forming or converting enzymes.^14^ During the four rounds of evolution, one colony was selected for sequencing after 6 days of incubation in the first round, which revealed the mutations S286R/S287T occurred in KdcA. In the second and third rounds, three colonies were sequenced after 5 days of incubation, yielding KdcA variants carrying the mutations S286R/S287T/F381H/F382P and S286R/S287T/F381H/F382P/M538T/G539F, respectively. In the fourth round, five colonies were ultimately grown on M9 agar plates after 4 days of incubation (Supplementary Figure 3C). Two large colonies were selected for sequencing, which showed that both harbored a KdcA variant with the following amino acid changes: S286R/S287T/F381H/F382P/L534S/L535F/M538T/G539F (referred to as KdcA^M8^). To further validate these findings, a confirmatory screen was performed by mixing the identified colonies from each round of selection with the BL21­(DE3) Δ*panD*/pKdcA-BAPAT strain, followed by dilution and plating on M9 agar plates for 5 days of cultivation (Supplementary Figure 3D). Five large colonies were selected for sequencing, and all carried the KdcA^M8^ variant. Subsequently, BL21­(DE3) Δ*panD* strains harboring KdcA variants identified from the four rounds of evolution, along with BAPAT, were cultivated in the M9 liquid medium for 5 days. Apparently, the growth of BL21­(DE3) Δ*panD* strains in rounds of evolution roughly correlates with the accumulation of mutations. The most pronounced growth was observed for the strain harboring the octuple KdcA variant (KdcA^M8^), compared to strains containing the double, quadruple, and sextuple KdcA variants (Supplementary Table 4 and Figure 3E). In addition, the protein expression of KdcA^M8^ was analyzed by SDS-PAGE, which demonstrates that, even though the KdcA^M8^ mutant exhibits a visible target band in protein expression, its insoluble fraction is highly dominant (Supplementary Figure 4). Thus, the reduced soluble expression of KdcA^M8^ compared to the WT KdcA could be one of the issues that impairs its *in vivo* activity, resulting in the slow growth of the variant in the minimal medium.

**Figure 2:**
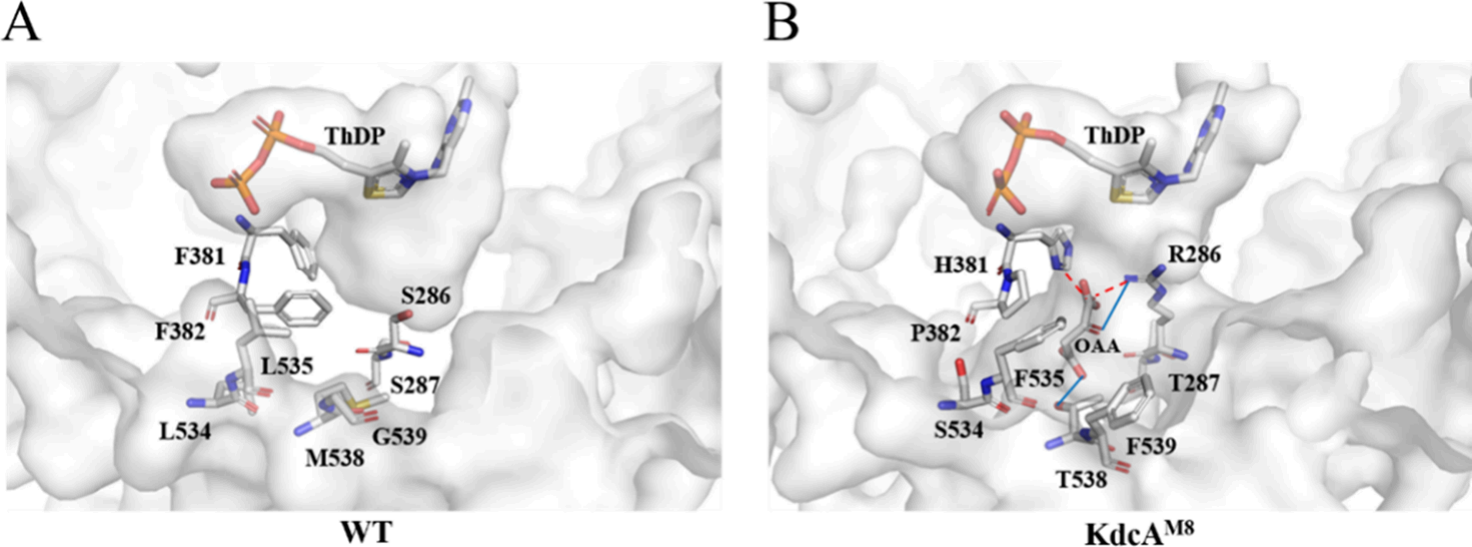
Surface structure and binding pocket of WT KdcA and KdcA^M8^. (A) Selected target sites for mutagenesis in the binding pocket of WT KdcA (PDB: 2VBG). (B) Docking of oxaloacetate in the binding pocket of the KdcA^M8^ structure. Solid blue lines represent hydrogen bonds, while red dotted lines indicate salt bridges. Molecular docking was performed using AutoDock Vina, and the docking results were analyzed using PLIP (Protein–Ligand Interaction Profiler). ThDP: thiamine pyrophosphate; OAA: oxaloacetate.

### Expression and Characterization of KdcA Variants

Protein engineering of KdcA through directed evolution resulted in the obtainment of the KdcA^M8^ variant. The *K*_M_ values of both WT KdcA and KdcA^M8^ were subsequently measured. Using a whole-cell catalyst, KdcA^M8^ exhibited a *K*_M_ of 1.15 mM (Supplementary Figure 7), whereas the *K*_M_ of WT KdcA exceeded 25 mM, as the enzyme remained unsaturated even at oxaloacetate concentrations as high as 50 mM (Supplementary Table 5). Previous studies have shown that *K*_M_ values determined using whole-cell catalysts are often higher than those obtained from lysates or purified enzymes, due to the diffusion barrier imposed by the cell membrane.^15,16^ Although the *K*_M_ value of KdcA^M8^ was measured under the less favorable conditions in whole cells, KdcA^M8^ still showed a lower *K*_M_ compared to that of WT KdcA as measured under more favorable conditions in lysate. This strongly suggests that KdcA^M8^ has a higher affinity for oxaloacetate than WT KdcA.

To explore the molecular basis for this enhanced substrate affinity, a homology model of KdcA^M8^ was constructed using AlphaFold2 based on the X-ray structure of KdcA. Oxaloacetate was then docked into this model. Upon structural alignment of WT KdcA and KdcA^M8^ (Supplementary Figure 8), the mutations S286R and S287T apparently reduce the size of the binding pocket as the side chains are enlarged and orient inward toward the center of the pocket. In contrast, the mutations F381H and F382P are positioned away from the pocket center and appear to create additional space within the binding pocket to accommodate the α/β-carbonyl groups of oxaloacetate. Mutations at the C-terminal helix, including L534S, L535F, M538T, and G539F, shift the C-terminal helix away from the binding pocket due to steric hindrance (Supplementary Figure 9), particularly from the introduction of bulkier phenylalanine residues at positions 535 and 539. This shift results in the displacement of F542 from the binding pocket, thereby opening the substrate channel. In WT KdcA, it was noted that F542 blocks the entrance to the active site, restricting substrate access.^17^ Furthermore, among these mutations, S286R provides a positively charged side chain that forms a salt bridge and a hydrogen bond with the α-carbonyl and α-keto groups of oxaloacetate, respectively. F381H provides a salt bridge between the imidazole ring and the α-carbonyl group of oxaloacetate, while M538T forms a hydrogen bond with the β-carbonyl group of oxaloacetate (Figure 2B). Overall, these potential new interactions between the substrate and mutated residues can contribute to the improved affinity of KdcA^M8^ toward oxaloacetate.

To evaluate the impact of each mutation on KdcA^M8^, several KdcA variants were created through site-directed mutagenesis, including S286R, S287T, F381H, F382P, L534S, L535F, M538T, and G539F. The decarboxylation activity of these variants was subsequently measured *in vitro*. According to the results shown in Figure 3, the S286R variant exhibited a catalytic activity of 0.011 μmol min^–1^ mg^–1^ lysate protein toward oxaloacetate, which is approximately 5 times higher than the activity of WT KdcA (0.002 μmol min^–1^ mg^–1^ lysate protein), without a significant change in protein expression levels (Supplementary Figure 5). According to the docking results shown in Supplementary Figure 10, S286 forms a hydrogen bond with the ketone group of oxaloacetate in WT KdcA. In contrast, substituting S286 with arginine, a positively charged residue with a large side chain, resulted in the formation of a salt bridge with the carboxyl group of oxaloacetate in the S286R variant. This salt bridge represents a significantly stronger interaction than a hydrogen bond. Therefore, it is reasonable to assume that this substitution might facilitate the anchoring of the substrate oxaloacetate in the binding pocket in a favorable orientation for catalysis, enhancing the enzymatic activity. Substituting S287 with threonine led to a 30% enhancement in activity compared to WT KdcA. Previous studies have suggested that shaping the binding pocket of KdcA and its homologue KivD can influence both catalytic activity and substrate preference.^18,19^ For example, replacing S286 with leucine in KdcA enhanced its catalytic activity toward 2-keto-3-deoxy--xylonic acid by reducing the size of the substrate-binding pocket.^18^ Similarly, replacing S286 with threonine in KivD decreased the pocket size, leading to improved catalytic activity and an increased isobutanol-to-isopentanol molar ratio in *Synechocystis* PCC 6803.^19^ Since S287 was next to S286, the improved activity of the S287T variant toward oxaloacetate could also be attributed to the reduced size of the binding pocket. For the F381H, L534S, M538T, and G539F variants, catalytic activity was slightly lower compared to that of WT KdcA. In contrast, the F382P and L535F variants exhibited markedly reduced activity, with levels falling below 0.001 μmol min^–1^ mg^–1^ lysate protein (Supplementary Figure 11). Previous studies have indicated that residues such as S286, F381, and M538 play crucial roles in shaping the substrate-binding pocket, thereby influencing both enzyme activity and substrate selectivity.^13^ Herein, the functional impacts of mutations at positions S287, F382, L534, L535, and G539 suggest that these residues may also play important functional roles within the binding pocket. Notably, although the F382P and L535F variants were expressed at considerable levels (Supplementary Figure 5), both exhibited substantial loss of activity. This loss may be due to incomplete folding, where the proteins remain soluble but adopt conformations in which the active site is too perturbed to support enzymatic function.^20^ Interestingly, the S286R/F382P/G539F triple mutant exhibited enhanced activity (0.003 μmol min^–1^ mg^–1^ lysate protein; Supplementary Figure 11), exceeding that of WT KdcA as well as the individual F382P and G539F variants. These findings suggest that specific combinations of mutations can compensate for individual deleterious effects and lead to enhanced enzymatic activity. In the case of the S286R/F382P/G539F triple mutant, this compensatory effect may result from the stabilizing or reconfiguring influence of the S286R and G539F mutations on the active site architecture, which could alleviate the detrimental structural impact of the F382P mutation.

**Figure 3:**
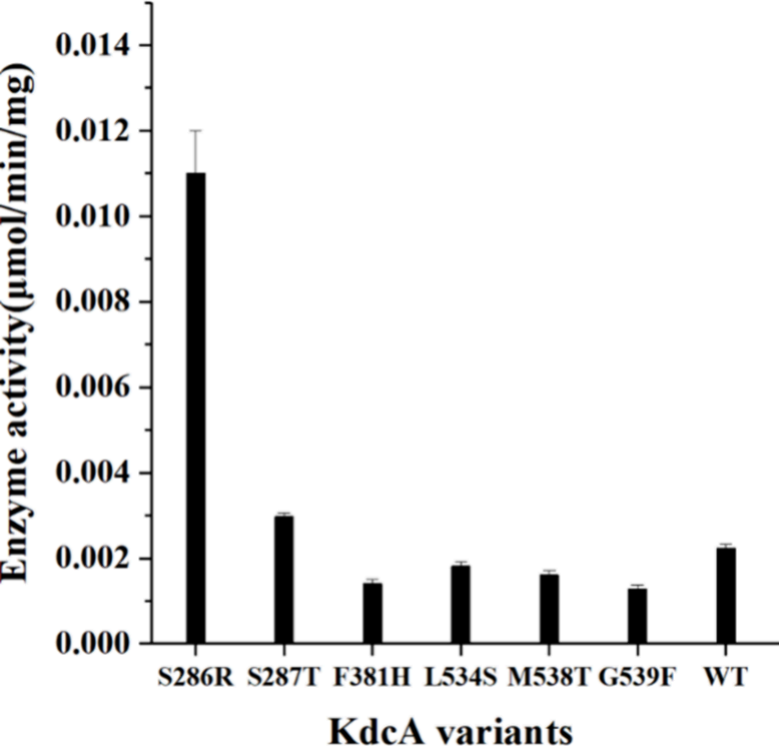
Decarboxylase activity of KdcA variants toward oxaloacetate, measured using a coupled enzymatic assay with a lysate protein as the biocatalyst.

### Construction of the Oxaloacetate Pathway for Biosynthesis of 3-HP in *E. coli*

To construct and explore the oxaloacetate pathway for the biosynthesis 3-HP in *E. coli*, the strain BP1 was developed, containing KdcA and 3-hydroxyisobutyrate dehydrogenase (HIBADH) from *P. putida* under the control of the T7 promotor, respectively. Expression of both proteins was successful (Supplementary Figure 6), while, during 48 h of cultivation, the 3-HP production level was disappointing (Figure 4B).

**Figure 4:**
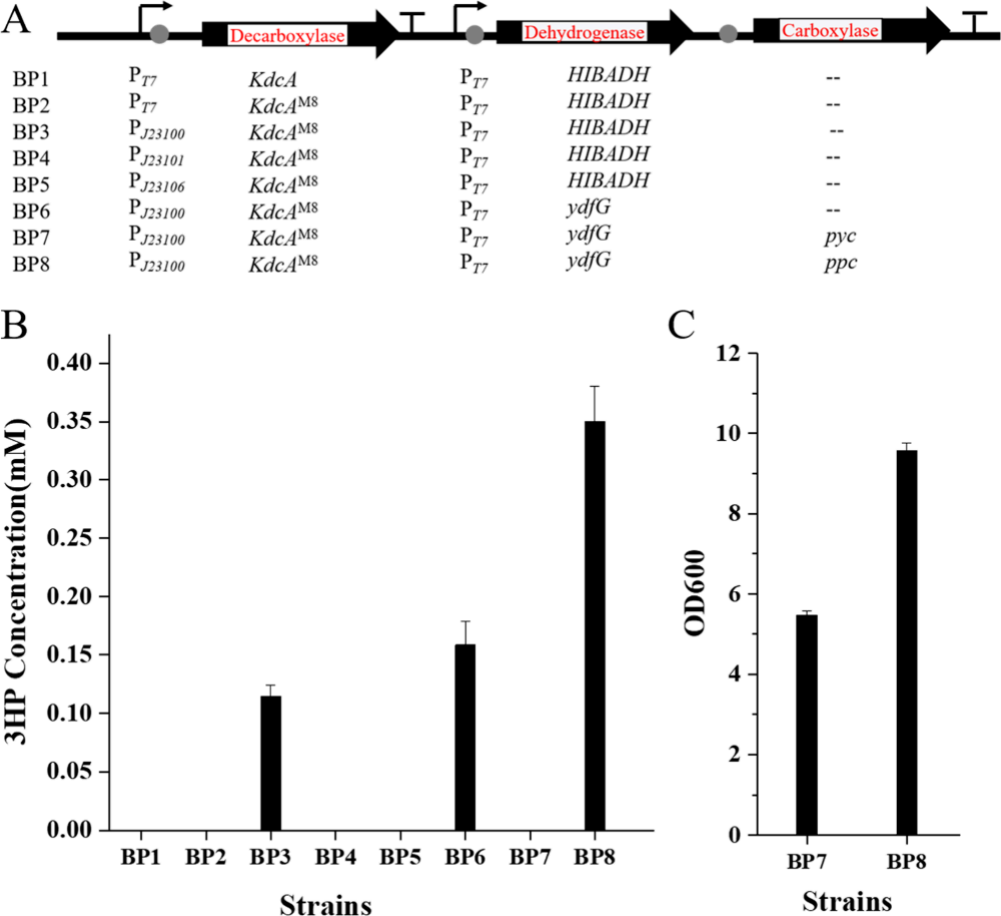
Construction of the oxaloacetate pathway for biosynthesis of 3-HP in *E. coli*. (A) A variety of plasmids were developed and utilized to construct the oxaloacetate pathway in strains. (B) *De novo* synthesis of 3-HP was achieved through enzyme screening and regulation of enzyme expression. (C) Cell density of BP7 and BP8 at 48 h.

Through protein engineering of KdcA, the KdcA^M8^ variant was developed and subsequently tested in the oxaloacetate pathway in place of WT KdcA that was used in BP1. To evaluate its performance, strains BP2, BP3, BP4, and BP5 were constructed, incorporating KdcA^M8^ under the control of promoters T7, J23100, J23101, and J23106, respectively. These constitutive promoters, obtained from the iGEM Parts Registry (http://parts.igem.org), were chosen for their distinct transcription rates,^21^ which allows to regulate KdcA^M8^ expression over the time of culture growth. Detailed promoter sequences are provided in Supplementary Table 3. The enzyme expression was monitored with SDS-PAGE, including the formation of inclusion bodies that can impair 3-HP production (Supplementary Figure 6). After 48 h of cultivation, strain BP3, which utilized the J23100 promoter to regulate KdcA^M8^ expression, achieved a production level of approximately 0.11 mM 3-HP (Figure 4B and Supplementary Figure 12), in contrast to BP2, BP4, and BP5. In particular, for strain BP2, where KdcA^M8^ expression is under the control of the T7 promoter, pronounced formation of inclusion bodies was observed, which matches the weak SDS-PAGE signal for the soluble target (Supplementary Figure 6). Since J23101 and J23106 are weaker promoters compared to J23100, overall expression levels for BP4 and BP5 were lower than those for BP3 (Supplementary Figure 6). These fermentation results indicate that the use of the J23100 promoter to regulate KdcA^M8^ expression effectively ensured protein expression and catalytic activity from the soluble fraction, alleviating inclusion body formation and enabling 3-HP production in BP3. The enhanced soluble expression of KdcA^M8^ driven by the J23100 promoter can be attributed to its relatively weak strength, which slows the protein synthesis rate, allowing more time for proper protein folding and increasing the amount of soluble, functional protein. The modulation of expression is commonly used to overcome solubility limitations.^22,23^ Since optimizing the soluble expression of KdcA^M8^ is critical for its application in 3-HP biosynthesis, future efforts to enhance 3-HP production should focus on improving KdcA^M8^ solubility. Potential strategies include coexpressing molecular chaperones, lowering the induction temperature, or fusing solubility-enhancing tags.^24^

In addition, replacing the T7 promoter with J23100 in strain BL21­(DE3) Δ*panD* significantly improved cell growth during 5 days of cultivation, as indicated by the increased cell density (OD600) (OD600 = 1.13 vs OD600 = 0.34; Supplementary Figure 3E). This further supported the critical role of J23100 in regulating the protein expression for enzymatic function. Moreover, the successful production of 3-HP using KdcA^M8^ instead of WT KdcA provides evidence that WT KdcA is a rate-limiting enzyme in the oxaloacetate pathway. Compared to WT KdcA, KdcA^M8^ demonstrated significantly improved affinity toward oxaloacetate, with a more than 25-fold decrease in the *K*_M_ value. This enhanced affinity may play a crucial role in enabling its application in 3-HP biosynthesis via the oxaloacetate pathway.

Besides HIBADH, 3-hydroxy acid dehydrogenase (YdfG) from *E. coli* is another candidate enzyme that catalyzes the dehydrogenation of malonic semialdehyde and is widely used in 3-HP production.^25^ Strain BP6 was engineered from BP3 by replacing HIBADH with YdfG to enhance 3-HP production. Upon cultivation, strain BP6 produced 0.16 mM 3-HP, representing a 45.4% increase compared to BP3 (Figure 4B and Supplementary Figure 12). These results demonstrate that combining KdcA^M8^ with YdfG provides an effective approach to 3-HP production. Furthermore, strain BPX was derived from BP6 by replacing KdcA^M8^ with the WT KdcA. In contrast to BP6, 3-HP production was undetectable in BPX (Supplementary Figure 12), confirming that the directed evolution of KdcA enhanced the biosynthetic efficiency of 3-HP production.

### Improving 3-HP Production through Enhancing the Precursor Supply and Culture Optimization

For 3-HP production via the oxaloacetate pathway, enhancing the metabolic flux toward oxaloacetate can represent a promising strategy to improve 3-HP synthesis. Previous studies have shown that overexpression of phosphoenolpyruvate carboxylase gene (*ppc*) and pyruvate carboxylase gene (*pyc*) can effectively increase oxaloacetate supply in *E. coli*.^26–29^ Based on this insight, strains BP7 and BP8 were engineered to increase 3-HP production by enhancing oxaloacetate levels through the overexpression of the *pyc* gene from *L. lactis* in BP7 and the *ppc* gene from *E. coli* in BP8. Strain BP8 accumulated 0.35 mM 3-HP, representing an 118% increase compared to the parental strain BP6 (Figure 4B and Supplementary Figure 12). This improvement indicates that the introduction and overexpression of the *ppc* gene effectively enhanced the oxaloacetate supply, leading to a significant increase in the level of 3-HP production. In contrast, strain BP7 exhibited impaired growth during 48 h of cultivation with a significantly lower optical density (5.4) compared to BP8 (9.5) (Figure 4C). This observation suggests that overexpression of the *pyc* gene can disrupt cellular metabolism, thereby affecting cell growth and interfering with 3-HP biosynthesis in BP7. Since enhancing the supply of oxaloacetate proved to be highly beneficial for 3-HP production in BP8, it is meaningful to explore additional strategies in future work to further increase oxaloacetate availability. These strategies may include promoting the glyoxylate shunt by deleting the *iclR* gene, weakening the TCA cycle by disrupting the *gltA* gene, or reducing PEP consumption by deleting the *pykF* gene.^30–32^

To further enhance 3-HP production in BP8, medium composition and induction conditions were optimized, including adjustments to glucose and yeast extract concentrations, the inducer isopropyl-β--1-thiogalactopyranoside (IPTG) concentration, and the addition of sodium bicarbonate (NaHCO_3_). As shown in Figure 5A,B, the optimal conditions involved 20 g/L glucose and 0.1 mM IPTG. Increasing the yeast extract concentration to 10 g/L further improved the 3-HP titer to 0.48 mM (Figure 5C). Moreover, the addition of 20 mM sodium bicarbonate (NaHCO_3_) to the culture medium resulted in a final 3-HP production of 0.71 mM (Figure 5D and Supplementary Figure 13). Previous studies have shown that the addition of carbonate compounds, such as MgCO_3_ or NaHCO_3_, can enhance succinate production in *E. coli*. This enhancement was attributed to the increased availability of CO_2_ derived from these compounds, which can stimulate the activity of PPC and consequently enhance carbon flux toward succinate biosynthesis.^33,34^ In this study, the observed increase in the 3-HP titer in BP8 following NaHCO_3_ supplementation may be explained by a similar mechanism. As HCO_3_^–^ is the direct substrate of PPC, its elevated intracellular availability is believed to enhance CO_2_ fixation by PPC.^35–37^ This, in turn, promotes carbon flux toward oxaloacetate biosynthesis, ultimately contributing to the increased production of 3-HP.

**Figure 5:**
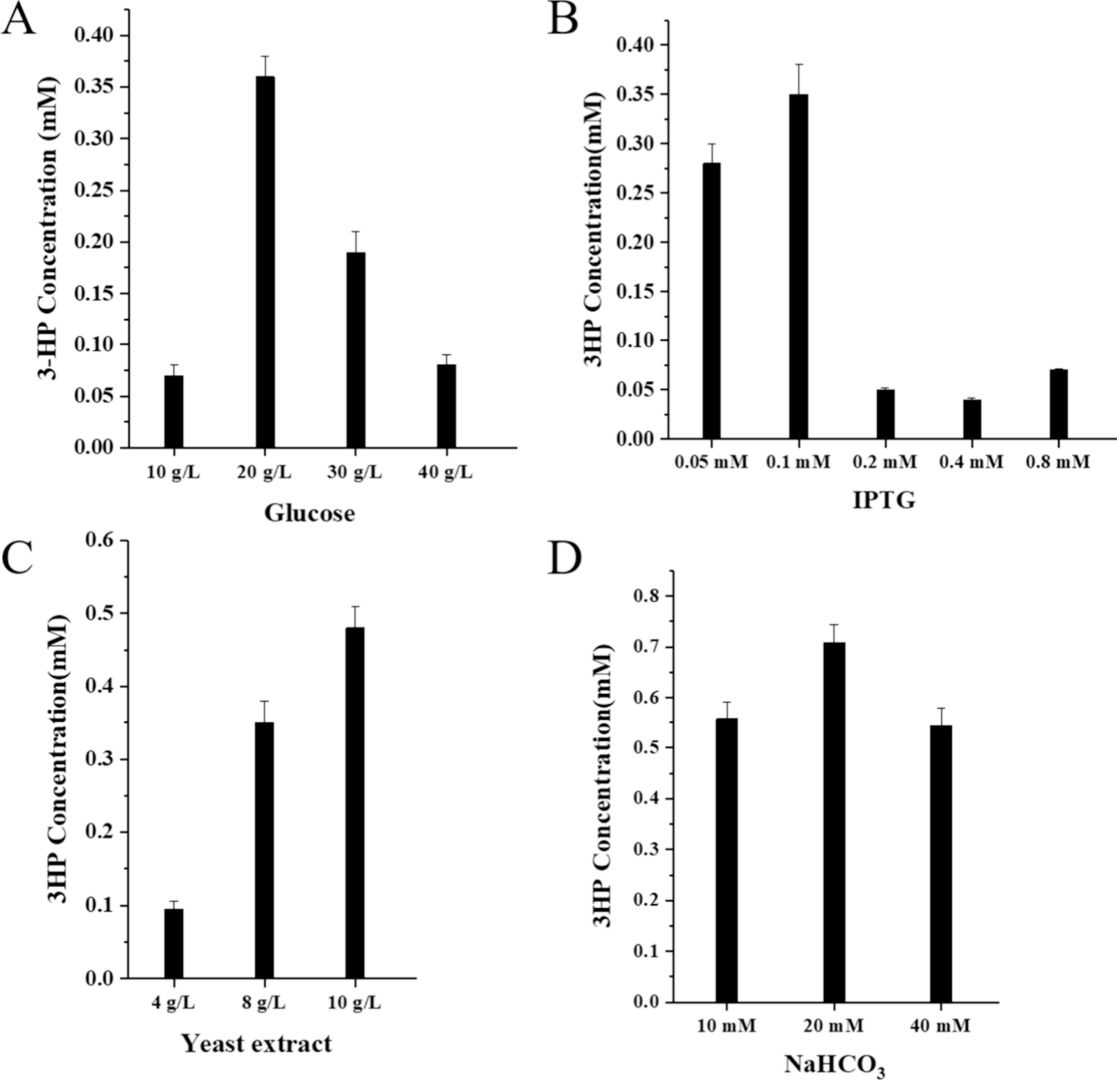
Improving 3-HP production by optimizing the culture parameters. (A) 3-HP production with different concentrations of glucose. (B) 3-HP production with different concentrations of IPTG. (C) 3-HP production with different concentrations of yeast extract. (D) 3-HP production with different concentrations of NaHCO_3_, which was added after 2 h of induction.

As shown in Table 1, the 3-HP yield (0.006 mol/mol) achieved via the oxaloacetate pathway in *E. coli* was significantly lower than that obtained using the malonyl-CoA pathway (0.031 mol/mol) and the β-alanine pathway (0.846 mol/mol) in *E. coli*,^38,39^ as well as the oxaloacetate pathway in *S. cerevisiae* (0.25 mol/mol).^7^ In the malonyl-CoA and β-alanine pathways, native or well-characterized enzymes were employed, facilitating efficient pathway construction for the 3-HP biosynthesis. In contrast, the oxaloacetate pathway depends on the engineered enzyme KdcA^M8^ to catalyze the rate-limiting step of converting oxaloacetate into malonic semialdehyde. However, the low soluble expression of KdcA^M8^ in *E. coli* impaired its catalytic activity toward oxaloacetate, thereby reducing the overall efficiency of the pathway and contributing to the observed low 3-HP yield.

**Table 1:** Representative Studies on Biotechnological 3-HP Production Using Different Chassis Strains and Pathways from Glucose Sources

**microorganism**	**pathway**	**engineering strategy**	**titer (mM)**	**yield** (mol/mol)	**ref.**
*E. coli*	malonyl-CoA pathway	expression of *mcr*, *accABCD*, and *pntAB*	2.14	0.031	^38^
*E. coli*	β-alanine pathway	expression of *pa0132*, *ydfG*, *panD*, *aspA*, and *ppc*; deletion of *fumAC*, *fumB*, and *iclR*	345.5	0.846	^39^
*S. cerevisiae*	oxaloacetate pathway	expression of *pyc*, *mdlC*, *mmsB*, *glc7*, and *ptc7*	201.1	0.25	^7^
*E. coli*	oxaloacetate pathway	expression of *KdcA* ^M8^ *ppc* and *ydfG*; optimization of culture conditions	0.71	0.006	this study

Additionally, in the application of the oxaloacetate pathway for 3-HP biosynthesis in *S. cerevisiae*, the host strain was specifically engineered to accumulate pyruvate, thereby enhancing oxaloacetate availability via pyruvate carboxylation.^7^ In contrast, this study relied solely on the overexpression of PPC to increase the oxaloacetate supply, without further engineering the *E. coli* host to redirect central carbon metabolism toward oxaloacetate biosynthesis. The inherent differences in metabolic flux between the two host strains likely also contributed to the disparity in 3-HP yields observed between *E. coli* and *S. cerevisiae*.

Despite the relatively low final titer and yield of 3-HP achieved in this study compared to other biosynthetic approaches, the oxaloacetate pathway offers distinct advantages. In this pathway, the oxaloacetate precursor is directly derived from PEP, whereas in the malonyl-CoA and β-alanine pathways, the biosynthesis of their respective precursors involves multiple enzymatic steps originating from PEP, leading to carbon flux loss. Furthermore, in the malonyl-CoA pathway, pyruvate serves as a core intermediate and is diverted not only toward malonyl-CoA formation but also into competing metabolic pathways such as acetate metabolism, fatty acid biosynthesis, and the glyoxylate cycle.^40,41^ This metabolic competition is believed to limit the carbon flux toward 3-HP production.^41–44^ In contrast, the oxaloacetate pathway could circumvent this issue by avoiding direct reliance on pyruvate, thereby offering a potentially efficient route for redirecting the carbon flux toward 3-HP biosynthesis.

## Conclusions

In this study, we demonstrated the biosynthesis of 3-HP via the oxaloacetate pathway in *E. coli*. To enhance the catalytic efficiency of KdcA toward oxaloacetate, protein engineering was performed and a growth selection system was adopted to isolate and identify positive KdcA variants from mutation libraries. After four rounds of selection, the variant KdcA^M8^, with mutations S286R/S287T/F381H/F382P/L534S/L535F/M538T/G539F in KdcA, was isolated. SDS-PAGE analysis revealed that KdcA^M8^ exhibits a decreased soluble expression. Using a whole-cell catalyst, KdcA^M8^ exhibited a significant improvement in affinity toward oxaloacetate compared to WT KdcA when measured in lysate (*K*_M_ = 1.15 mM vs *K*_M_ > 25 mM). To evaluate the contribution of each individual mutation in KdcA^M8^, we performed site-directed mutagenesis and *in vitro* activity assays were performed. The S286R and S287T variants showed 5.5-fold and 1.3-fold increases in activity, respectively, whereas the F381H, F382P, L534S, L535F, M538T, and G539F variants exhibited reduced activity compared to WT KdcA. These results suggest that, in addition to the previously identified key residues Ser286, Phe381, and Met538,^13^ other residues such as Ser287, Phe382, Leu534, Leu535, and Gly539 may also be functionally important within the substrate-binding pocket. Furthermore, combinatorial mutagenesis revealed that certain combinations of mutations can compensate for individual deleterious effects and lead to enhanced activity. Notably, the S286R/F382P/G539F triple mutant exhibited higher activity than that of WT KdcA, as well as the individual F382P and G539F variants.

In the biosynthesis of 3-HP, the low affinity of KdcA for oxaloacetate makes it a rate-limiting enzyme in the oxaloacetate pathway, effectively prohibiting the production of 3-HP production. In contrast, replacing WT KdcA with KdcA^M8^, which has improved affinity for oxaloacetate, enabled 3-HP biosynthesis via the oxaloacetate pathway in *E. coli*. Indicating that enhancing KdcA’s binding affinity for oxaloacetate is crucial for its application in 3-HP biosynthesis. By regulating KdcA^M8^ expression, optimizing dehydrogenase selection, increasing oxaloacetate supply, and improving culture conditions, 0.71 mM of 3-HP was successfully produced. Although the oxaloacetate pathway for 3-HP biosynthesis in *E. coli* was successfully established, the final 3-HP titer remains low. To further increase 3-HP production, strategies could be adopted, such as enhancing the activity and soluble expression of KdcA^M8^, as well as increasing oxaloacetate flux.

## Materials and Methods

### Strains and Plasmids

All strains and plasmids utilized in this research are detailed in Supplementary Table 1. *E. coli* DH5α was employed for gene cloning, while BL21­(DE3) served as the host strain for protein expression and 3-HP production. The plasmid pET28a was utilized for the overexpression of genes associated with the 3-HP synthesis module. Strain BL21­(DE3) Δ*panD* was used as the host in the directed evolution experiments.

### Plasmid Construction

DNA fragments were amplified using a Phusion Plus DNA Polymerase. Gel purifications of all DNA products were performed using the GeneJET Gel Extraction Kit. Plasmid extractions were conducted by using the GeneJET Plasmid Miniprep Kit. These items were purchased from Thermo Fisher Scientific.

The primers were used for constructing plasmids, as detailed in Supplementary Table 2. All fragments were inserted into the plasmid pET28a using the Gibson assembly. To construct p*K*, the *KdcA* gene from *L. lactis* was amplified from the genome and inserted into pET28a under the control of a T7 promoter. For plasmid pH, the *HIBADH* gene from *P. putida* KT2400 was amplified from the genome and inserted into pET28a under the control of a T7 promoter. To construct pKB, the *BAPAT* gene from *P. putida* KT2400 and the *KdcA* gene from p*K* were amplified. Subsequently, the *KdcA* and *BAPAT* fragments were inserted into the pET28a plasmid under the control of T7 promoters, respectively. Plasmid pKH was constructed by inserting the *KdcA* and *HIBADH* fragments into the pET28a plasmid under the control of the T7 promoters. p*K* ^M8^H was constructed by replacing the *KdcA* gene of pKH with *KdcA* ^M8^. Plasmids p100 K^M8^H, p101 K^M8^H, and p106 K^M8^H were constructed by replacing the T7 promoter of p*K* ^M8^H with J23100, J23101, and J23106 promoters, respectively. These synthetic promoters are listed in Supplementary Table 3. Plasmid p100 K^M8^Y was constructed by replacing the *HIBADH* gene of p100 K^M8^H with the *ydfG* gene from *E. coli*. To construct p100 K^M8^YY and p100 K^M8^YP, the *pyc* gene and the *ppc* gene were amplified from the genomes of *L. lactis* and *E. coli*, respectively. These genes were then inserted after the *ydfG* gene in p100 K^M8^Y.

### Knockout of the *panD* Gene

The BL21­(DE3) Δ*panD* strain was engineered by knocking out the *panD* gene from the genome using CRISPR-Cas9 technology.^45,46^ Plasmid pEcCas integrates the arabinose-inducible λ-Red recombineering system, a Cas9 expression system, and a plasmid curing system into a single vector. The sgRNA donor plasmid, pEcgRNA-*panD*, was constructed by PCR amplifying the pEcgRNA backbone using primers N20-*panD*-F and N20-*panD*-R, followed by self-ligation of the PCR product. The donor double-stranded DNA (dsDNA), containing 500 bp homology arms upstream and downstream of the target gene, was generated via fusion PCR using primers listed in Supplementary Table 2.

To construct *E. coli* BL21 strains harboring pEcCas, 50 μL of competent cells was electroporated with 10 ng of pEcCas using a 1 mm Gene Pulser cuvette (Bio-Rad) at 1.8 kV, 25 μF, and 200 Ω. The cells immediately recovered in 300 μL of fresh LB medium and incubated at 37 °C with shaking at 220 rpm for 1 h. The culture was then plated on LB agar containing kanamycin (50 μg/mL) and incubated overnight at 37 °C. A single colony was picked and cultured overnight in 4 mL of LB with kanamycin (50 μg/mL) to prepare electrocompetent cells, following the procedure described in a previous study.^46^

For genome editing, 50 μL of the prepared electrocompetent cells was electroporated with 50 ng of pEcgRNA-*panD* and 100 ng of donor dsDNA using the same conditions as above. Cells were recovered in 300 μL of LB at 30 °C for 1 h and then plated on LB agar containing kanamycin (50 μg/mL) and spectinomycin (50 μg/mL), followed by overnight incubation at 30 °C. Colony PCR was performed to screen and verify positive colonies, using wild-type strains as controls (Supplementary Figure 1).

To eliminate the pEcgRNA-*panD* plasmid, positive colonies were inoculated into 2 mL of LB medium containing 10 mM rhamnose and kanamycin and incubated overnight at 37 °C, 220 rpm. The culture was then plated on LB agar with kanamycin, and colonies sensitive to spectinomycin were considered to be cured of pEcgRNA-*panD*. For curing the pEcCas plasmid, the spectinomycin-sensitive colonies were inoculated into the LB medium containing glucose (5 g/L) and grown overnight at 37 °C, 220 rpm. Cultures were then plated on LB agar supplemented with glucose (5 g/L) and sucrose (10 g/L) and incubated overnight at 37 °C. Colonies that became sensitive to kanamycin were confirmed to be cured of pEcCas.

### Directed Evolution

For the evolution of KdcA, mutation libraries were created through multiple site-saturation mutagenesis with the Q5 Site-Directed Mutagenesis Kit (New England Biolabs), using pKB as the template. The specific primers used are detailed in Supplementary Table 2.

The BL21­(DE3) Δ*panD* cells were transformed with the mutation library by electroporation using the same conditions as above. Immediately after the electroporation pulse, 1 mL of prewarmed LB medium (37 °C) was added to the cuvette, and the cells were incubated at 30 °C and 200 rpm for 2 h. A 30 μL aliquot of the culture was taken to assess electroporation efficiency, while the remaining cells were centrifuged, washed twice with water, and plated on M9 agar (lacking yeast extract) supplemented with 4 mg/L ThDP, 2 mg/L pyridoxal 5′-phosphate, and 25 μg/mL kanamycin. After 4–6 days of incubation at 30 °C, large colonies were selected and streaked onto fresh plates.

### Kinetic Parameters and Enzyme Activities of KdcA Variants

The enzymatic activities of KdcA variants were measured using a coupled assay with an excess of HIBADH. The assay mixture, prepared in a 100 μL volume, contained 20 mM oxaloacetate, 5 mM NADH, 2 mM MgSO_4_, 0.5 mM ThDP, 1 mg/mL purified HIBADH, and 40 μL of lysate protein in a 50 mM potassium phosphate buffer (pH 6.5). The reaction was initiated by adding the lysate protein and incubated at 30 °C for 1 h, followed by analysis via HPLC. To obtain the lysate protein, KdcA variants were expressed in *E. coli* BL21­(DE3) using 0.1 mM IPTG at 30 °C for 3 h. Cells were then harvested by centrifugation at 4 °C, washed twice, resuspended in a 50 mM PBS solution (pH 6.5) to adjust OD600 to 4.0, and disrupted by ultrasonication. The lysate protein was obtained as the supernatant after centrifugation.

The *K*_M_ of WT KdcA was determined by measuring reaction rates using oxaloacetate concentrations ranging from 1 to 50 mM, with 5 mM NADH under the same assay conditions described above, using the lysate protein as the biocatalyst. For the KdcA^M8^ variant, the *K*_M_ was similarly determined using oxaloacetate concentrations ranging from 0 to 20 mM and 5 mM NADH, but using 80 μL of whole-cell biocatalyst in place of lysate protein and purified HIBADH. To obtain the whole-cell biocatalyst, strain BP3, coexpressing KdcA^M8^ and HIBADH, was cultivated in a modified M9 medium and induced with 0.1 mM IPTG at 30 °C for 12 h. Cells were then harvested, washed twice, and resuspended in a 50 mM PBS solution (pH 6.5) to adjust the OD600 to 10.0.

### 3-HP Production in Shaking Flasks

For the 3-HP production experiments, a single colony was inoculated into 4 mL of LB medium (containing 10 g/L tryptone, 5 g/L yeast extract, and 10 g/L NaCl) and incubated overnight at 37 °C. The cells grown in LB were then transferred to 250 mL flasks with 20 mL of modified M9 medium. The cultivation was performed in a shaking incubator at 220 rpm and 37 °C. When the OD600 reached 0.8, 0.1 mM isopropyl β--1-thiogalactopyranoside (IPTG) was added, and the temperature was adjusted to 30 °C. All flask cultivations were performed in duplicate.

The modified M9 medium contained the following per liter: glucose, 20 g; yeast extract, 10 g; Na_2_HPO_4_, 7 g; KH_2_PO_4_, 3 g; NaCl, 0.5 g; NH_4_Cl, 1 g; MgSO_4_·7H_2_O, 246 mg; CaCl_2_·2H_2_O, 14.7 mg; thiamine, 1 mg; biotin, 1 mg; ThDP 4 mg; and a trace element solution, 10 mL. The trace element solution contained the following per liter: EDTA, 5 g; FeCl_3_·6H_2_O, 0.83 g; ZnCl_2_, 84 mg; CuCl_2_·2H_2_O, 13 mg; CoCl_2_·2H_2_O, 10 mg; H_3_BO_3_, 10 mg; and MnCl_2_·4H_2_O, 1.6 mg. Kanamycin (50 μg/mL) was also added.

### Analytical Procedures

The yield of 3-HP was determined with an analytical UltiMate 3000 HPLC system (Thermo Fisher Scientific). The analysis was conducted by using a Bio-Rad Aminex HPX-87H column (Bio-Rad Laboratories) with RID and UV detectors. The column was maintained at a temperature of 14 °C, and 5 mM sulfuric acid flowing at a rate of 0.4 mL/min was used as the mobile phase. The biomass was quantified by measuring the turbidity of the culture medium at 600 nm by using a spectrophotometer.

## Supplementary Material



## Notes

Detailed methods of strains, plasmids, and promoter sequences used in the study (Supplementary Tables 1–3); summary of KdcA mutations and the data of WT KdcA activity (Supplementary Tables 4 and 5); verification of *panD* gene deletion (Supplementary Figures 1); growth performance of *E. coli* BL21­(DE3) Δ*panD* with or without a complementary β-alanine pathway (Supplementary Figures 2); screening of KdcA variants from mutant libraries and the corresponding growth performance of the identified KdcA variant (Supplementary Figures 3); protein expression of KdcA variants (Supplementary Figures 4–6); kinetic parameters of KdcA^M8^ variant (Supplementary Figure 7); structural comparison and protein–ligand interaction analysis of KdcA variants (Supplementary Figures 8–10); HPLC analysis of 3-HP during enzyme activity assays and in shaking flask culture (Supplementary Figures 11–13); and DNA sequences of the following genes: *kdcA* from *L. lactis*, *HIBADH* from *P. putida* KT2400, *ydfG* and *ppc* from *E. coli*, and *pyc* from *L. lactis* (PDF)

## References

[1] Zhang F. Z., Rodriguez S., Keasling J. D. (2011). Metabolic engineering of microbial
pathways for advanced biofuels production. Curr.
Opin. Biotechnol..

[2] Lee J. W., Na D., Park J. M., Lee J., Choi S., Lee S. Y. (2012). Systems
metabolic engineering of microorganisms for natural and non-natural
chemicals. Nat. Chem. Biol..

[3] Vidra A., Németh A. (2018). Bio-based
3-hydroxypropionic Acid: A Review. Periodica
Polytechnica-Chemical Engineering.

[4] Valdehuesa K. N. G., Liu H. W., Nisola G. M., Chung W. J., Lee S. H., Park S. J. (2013). Recent advances
in the metabolic engineering of microorganisms
for the production of 3-hydroxypropionic acid as C3 platform chemical. Appl. Microbiol. Biotechnol..

[5] Jiang X. L., Meng X., Xian M. (2009). Biosynthetic
pathways for 3-hydroxypropionic
acid production. Appl. Microbiol. Biotechnol..

[6] Della
Pina C., Falletta E., Rossi M. (2011). A green approach to chemical building
blocks. The case of 3-hydroxypropanoic acid. Green Chem..

[7] Tong T., Tao Z. Y., Chen X. L., Gao C., Liu H., Wang X. L., Liu G. Q., Liu L. M. (2021). A biosynthesis pathway
for 3-hydroxypropionic acid production in genetically engineered *Saccharomyces cerevisiae*. Green Chem..

[8] Zeymer, C. ; Hilvert, D. , Directed Evolution of Protein Catalysts. In Annual Review of Biochemistry, Kornberg, R. D. , Ed. 2018; Vol. 87, pp 131–157. 10.1146/annurev-biochem-062917-012034 29494241

[9] Hammer S. C., Knight A. M., Arnold F. H. (2017). Design
and evolution of enzymes for
non-natural chemistry. Current Opinion in Green
and Sustainable Chemistry.

[10] Wang Y. J., Xue P., Cao M. F., Yu T. H., Lane S. T., Zhao H. M. (2021). Directed
Evolution: Methodologies and Applications. Chem.
Rev..

[11] Yuan L., Kurek I., English J., Keenan R. (2005). Laboratory-directed
protein evolution. Microbiol. Mol. Biol. Rev..

[12] Liu C. S., Ding Y. M., Zhang R. B., Liu H. Z., Xian M., Zhao G. (2016). Functional balance
between enzymes in malonyl-CoA pathway for 3-hydroxypropionate
biosynthesis. Metabolic Engineering.

[13] Yep A., Kenyon G. L., McLeish M. J. (2006). Determinants
of substrate specificity
in KdcA, a thiamin diphosphate-dependent decarboxylase. Bioorganic Chemistry.

[14] Wu S. K., Xiang C., Zhou Y., Khan M. S. H., Liu W. D., Feiler C. G., Wei R., Weber G., Höhne M., Bornscheuer U. T. (2022). A growth
selection system for the directed evolution
of amine-forming or converting enzymes. Nat.
Commun..

[15] Tan S. I., Han Y. L., Yu Y. J., Chiu C. Y., Chang Y. K., Ouyang S., Fan K. C., Lo K. H., Ng I. S. (2018). Efficient
carbon dioxide sequestration by using recombinant carbonic anhydrase. Process Biochemistry.

[16] Desouza M. P., Yoch D. C. (1995). Purification and characterization
of dimethylsulfoniopropionate
lyase from an alcaligenes-like dimethyl sulfide-producing marine isolate. Appl. Environ. Microbiol..

[17] Berthold C. L., Gocke D., Wood D., Leeper F. J., Pohl M., Schneider G. (2007). Structure
of the branched-chain keto acid decarboxylase
(KdcA) from *Lactococcus lactis* provides insights
into the structural basis for the chemoselective and enantioselective
carboligation reaction. Acta Crystallographica
Section D-Structural Biology.

[18] Lv K. M., Cao X. F., Pedroso M. M., Wu B., Li J. H., He B. F., Schenk G. (2024). Structure-guided engineering
of branched-chain
α-keto acid decarboxylase for improved 1,2,4-butanetriol production
by *in vitro* synthetic enzymatic biosystem. Int. J. Biol. Macromol..

[19] Miao R., Xie H., M. Ho F., Lindblad P. (2018). Protein engineering of α-ketoisovalerate
decarboxylase for improved isobutanol production in *Synechocystis* PCC 6803. Metabolic Engineering.

[20] González-Montalbán N., García-Fruitós E., Villaverde A. (2007). Recombinant
protein solubility—does more mean better?. Nat. Biotechnol..

[21] Hu B. D., Yu H. B., Zhou J. W., Li J. H., Chen J., Du G. C., Lee S. Y., Zhao X. R. (2023). Whole-Cell P450
Biocatalysis Using Engineered *Escherichia coli* with
Fine-Tuned Heme Biosynthesis. Adv. Sci..

[22] Schumann W., Ferreira L. C. S. (2004). Production of recombinant proteins
in *Escherichia
coli*. Genetics and Molecular Biology.

[23] Kaur J., Kumar A., Kaur J. (2018). Strategies for optimization of heterologous
protein expression in *E. coli*: Roadblocks and reinforcements. Int. J. Biol. Macromol..

[24] Atroshenko D. L., Sergeev E. P., Golovina D. I., Pometun A. A. (2024). Additivities for
Soluble Recombinant Protein Expression in Cytoplasm of *Escherichia
coli*. Fermentation-Basel.

[25] Fujisawa H., Nagata S., Misono H. (2003). Characterization of
short-chain dehydrogenase/reductase
homologues of *Escherichia coli* (YdfG) and *Saccharomyces cerevisiae* (YMR226C). Biochimica Et Biophysica Acta-Proteins and Proteomics.

[26] Balzer G. J., Thakker C., Bennett G. N., San K. Y. (2013). Metabolic engineering
of *Escherichia coli* to minimize byproduct formate
and improving succinate productivity through increasing NADH availability
by heterologous expression of NAD^+^-dependent formate dehydrogenase. Metabolic Engineering.

[27] Song C. W., Lee J., Ko Y. S., Lee S. Y. (2015). Metabolic
engineering of *Escherichia coli* for the production
of 3-aminopropionic
acid. Metabolic Engineering.

[28] Lee K. H., Park J. H., Kim T. Y., Kim H. U., Lee S. Y. (2007). Systems
metabolic engineering of *Escherichia coli* for L-threonine
production. Mol. Syst. Biol..

[29] Cremer J., Eggeling L., Sahm H. (1991). Control of
the Lysine Biosynthesis
Sequence in *Corynebacterium glutamicum* as Analyzed
by Overexpression of the Individual Corresponding Genes. Appl. Environ. Microbiol..

[30] Liu M., Lou J. L., Gu J. L., Lyu X. M., Wang F. Q., Wei D. Z. (2020). Increasing L-homoserine
production in *Escherichia
coli* by engineering the central metabolic pathways. J. Biotechnol..

[31] Li Y. J., Wei H. B., Wang T., Xu Q. Y., Zhang C. L., Fan X. G., Ma Q., Chen N., Xie X. X. (2017). Current
status on metabolic engineering for the production of L-aspartate
family amino acids and derivatives. Bioresour.
Technol..

[32] Al
Zaid Siddiquee K., Arauzo-Bravo M. J., Shimizu K. (2004). Metabolic flux analysis
of *pykF* gene knockout *Escherichia coli* based on 13C-labeling experiments together with measurements of
enzyme activities and intracellular metabolite concentrations. Appl. Microbiol. Biotechnol..

[33] Wang D., Li Q., Li W. L., Xing J. M., Su Z. G. (2009). Improvement of succinate
production by overexpression of a cyanobacterial carbonic anhydrase
in *Escherichia coli*. Enzyme
Microb. Technol..

[34] Kwon Y. D., Kwon O. H., Lee H. S., Kim P. (2007). The effect of NADP-dependent
malic enzyme expression and anaerobic C4 metabolism in *Escherichia
coli* compared with other anaplerotic enzymes. J. Appl. Microbiol..

[35] Liu R. M., Liang L. Y., Wu M. K., Chen K. Q., Jiang M., Ma J. F., Wei P., Ouyang P. K. (2013). CO2 fixation for
succinic acid production by engineered *Escherichia coli* co-expressing pyruvate carboxylase and nicotinic acid phosphoribosyltransferase. Biochemical Engineering Journal.

[36] Vemuri G. N., Eiteman M. A., Altman E. (2002). Effects of
growth mode and pyruvate
carboxylase on succinic acid production by metabolically engineered
strains of *Escherichia coli*. Appl. Environ. Microbiol..

[37] Kai Y., Matsumura H., Izui K. (2003). Phosphoenolpyruvate carboxylase::
three-dimensional structure and molecular mechanisms. Arch. Biochem. Biophys..

[38] Rathnasingh C., Raj S. M., Lee Y., Catherine C., Ashok S., Park S. (2012). Production of 3-hydroxypropionic
acid via malonyl-CoA pathway using recombinant *Escherichia
coli* strains. J. Biotechnol..

[39] Song C. W., Kim J. W., Cho I. J., Lee S. Y. (2016). Metabolic Engineering
of *Escherichia coli* for the Production of 3-Hydroxypropionic
Acid and Malonic Acid through β-Alanine Route. ACS Synth. Biol..

[40] Krivoruchko A., Zhang Y. M., Siewers V., Chen Y., Nielsen J. (2015). Microbial
acetyl-CoA metabolism and metabolic engineering. Metabolic Engineering.

[41] Liu C. S., Ding Y. M., Xian M., Liu M., Liu H. Z., Ma Q. J., Zhao G. (2017). Malonyl-CoA pathway:
a promising
route for 3-hydroxypropionate biosynthesis. Critical Reviews in Biotechnology.

[42] Chen Y., Bao J. C., Kim I. K., Siewers V., Nielsen J. (2014). Coupled incremental
precursor and co-factor supply improves 3-hydroxypropionic acid production
in *Saccharomyces cerevisiae*. Metabolic Engineering.

[43] Liu M., Yao L., Xian M., Ding Y. M., Liu H. Z., Zhao G. (2016). Deletion of
arcA increased the production of acetyl-CoA-derived chemicals in recombinant *Escherichia coli*. Biotechnol. Lett..

[44] Rogers J. K., Church G. M. (2016). Genetically encoded sensors enable real-time observation
of metabolite production. Proc. Natl. Acad.
Sci. U. S. A..

[45] Li Q., Sun B. B., Chen J., Zhang Y. W., Jiang Y., Yang S. (2021). A modified pCas/pTargetF
system for CRISPR-Cas9-assisted genome editing
in *Escherichia coli*. Acta Biochimica
Et Biophysica Sinica.

[46] Shukal S., Lim X. H., Zhang C. Q., Chen X. X. (2022). Metabolic engineering
of *Escherichia coli* BL21 strain using simplified
CRISPR-Cas9 and asymmetric homology arms recombineering. Microb. Cell Factories.

