# Supplementary material for: Directed Evolution of Branched-Chain α‑Keto Acid Decarboxylase for 3‑Hydroxypropionic Acid Production in Escherichia coli via Oxaloacetate
Source: ACS Synth Biol. 2025 Aug 15;14(9):3487–96. doi: 10.1021/acssynbio.5c00267 (PMC12455653; doi:10.1021/acssynbio.5c00267)
Supplement: Supplementary file 1 [file sb5c00267_si_001.pdf]

Supporting information

Directed Evolution of Branched-chain α-Keto acid Decarboxylase for 3-Hydroxypropionic acid Production in Escherichia coli via Oxaloacetate

Chuang Wang[1], René C. L. Olsthoorn[1] and Huub J. M. De Groot[1]*

[1]Leiden Institute of Chemistry, Leiden University, 2300 RA Leiden, The Netherlands

*Corresponding author email: groot_h@lic.leidenuniv.nl

Supplementary Table 1. *E. coli* strains and plasmids were used in this study.

Strain/plasmid	Description	Source or reference
Strains		
E. coli DH5α	*F-supE44 ΔlacU169 (φ80 lacZΔM15) hsdR17 recA1 endA1 gyrA96 thi-1 relA1*	Invitrogen
E. coli BL21(DE3)	*F-ompT hsdSB (Rb-mB-) gal (λ c I 857 ind1 Sam7 nin5 lacUV5 T7gene1) dcm (DE3)*	Invitrogen
BL21(DE3) Δ*panD*	*Gene panD knock-out in E. coli* BL21(DE3)	This study
H0	*E. coli* BL21(DE3) containing plasmid pH	This study
B0	*E. coli* BL21(DE3) containing plasmid pK	This study
B1	*E. coli* BL21(DE3) containing plasmid pK1	This study
B2	*E. coli* BL21(DE3) containing plasmid pK2	This study
B3	*E. coli* BL21(DE3) containing plasmid pK3	This study
B4	*E. coli* BL21(DE3) containing plasmid pK4	This study
B5	*E. coli* BL21(DE3) containing plasmid pK5	This study
B6	*E. coli* BL21(DE3) containing plasmid pK6	This study
B7	*E. coli* BL21(DE3) containing plasmid pK7	This study
B8	*E. coli* BL21(DE3) containing plasmid pK8	This study

B9	*E. coli* BL21(DE3) containing plasmid pK9	This study
BP1	*E. coli* BL21(DE3) containing plasmid pKH	This study
BP2	*E. coli* BL21(DE3) containing plasmid pK^{M8}H	This study
BP3	*E. coli* BL21(DE3) containing plasmid p100K^{M8}H	This study
BP4	*E. coli* BL21(DE3) containing plasmid p101K^{M8}H	This study
BP5	*E. coli* BL21(DE3) containing plasmid p106K^{M8}H	This study
BP6	*E. coli* BL21(DE3) containing plasmid p100K^{M8}Y	This study
BP7	*E. coli* BL21(DE3) containing plasmid p100K^{M8}YY	This study
BP8	*E. coli* BL21(DE3) containing plasmid p100K^{M8}YP	This study
BPX	*E. coli* BL21(DE3) containing plasmid p100KY	This study
C1	*E. coli* BL21(DE3) Δ*panD* containing plasmid pB	This study
C2	*E. coli* BL21(DE3) Δ*panD* containing plasmid pK	This study
C3	*E. coli* BL21(DE3) Δ*panD* containing plasmid pKB	This study
C4	*E. coli* BL21(DE3) Δ*panD* containing plasmid pK^{M2}B	This study
C5	*E. coli* BL21(DE3) Δ*panD* containing plasmid pK^{M4}B	This study
C6	*E. coli* BL21(DE3) Δ*panD* containing plasmid pK^{M6}B	This study
C7	*E. coli* BL21(DE3) Δ*panD* containing plasmid pK^{M8}B	This study
C8	*E. coli* BL21(DE3) Δ*panD* containing plasmid p100K^{M8}B	This study
CX	*E. coli* BL21(DE3) containing plasmid pET28a(+)	This study
Plasmids		
pEcgRNA	*pMB1 aadA sgRNA ccdB, Spe*^{R}	Addgene
pEcgRNA-*panD*	Derived from pEcgRNA, target *panD* in *E. coli* BL21(DE3)	This study
pEcCas	*pSC101* P_{cas}-*cas9* P_{araB}-*Red lacIq* P_{rhaB}-*sgRNA-pMB1*, *Kan*^{R}	Addgene
pET28a(+)	*reppBR322 Kan*^{R} *lacI* P_{T7}	Lab stock

pB	*reppBR322 Kan*^{R} *lacI* P$_{T7}$ *BAPAT*	This study
pH	*reppBR322 Kan*^{R} *lacI* P$_{T7}$ *HIBADH*	This study
pK	*reppBR322 Kan*^{R} *lacI* P$_{T7}$ *KdcA*	This study
pK1	*reppBR322 Kan*^{R} *lacI* P$_{T7}$ *KdcA* S286R	This study
pK2	*reppBR322 Kan*^{R} *lacI* P$_{T7}$ *KdcA* S287T	This study
pK3	*reppBR322 Kan*^{R} *lacI* P$_{T7}$ *KdcA* F381H	This study
pK4	*reppBR322 Kan*^{R} *lacI* P$_{T7}$ *KdcA* F382P	This study
pK5	*reppBR322 Kan*^{R} *lacI* P$_{T7}$ *KdcA* L534S	This study
pK6	*reppBR322 Kan*^{R} *lacI* P$_{T7}$ *KdcA* L535F	This study
pK7	*reppBR322 Kan*^{R} *lacI* P$_{T7}$ *KdcA* M538T	This study
pK8	*reppBR322 Kan*^{R} *lacI* P$_{T7}$ *KdcA* G539F	This study
pK9	*reppBR322 Kan*^{R} *lacI* P$_{T7}$ *KdcA* S286R F381P G539F	This study
pKB	*reppBR322 Kan*^{R} *lacI* P$_{T7}$ *KdcA* P$_{T7}$ *BAPAT*	This study
pK^{M2}B	*reppBR322 Kan*^{R} *lacI* P$_{T7}$ *KdcA* S286R S287T P$_{T7}$ *BAPAT*	This study
pK^{M4}B	*reppBR322 Kan*^{R} *lacI* P$_{T7}$ *KdcA* S286R S287T F381H F382P P$_{T7}$ *BAPAT*	This study
pK^{M6}B	*reppBR322 Kan*^{R} *lacI* P$_{T7}$ *KdcA* S286R S287T F381H F382P M538T G539F P$_{T7}$ *BAPAT*	This study
pK^{M8}B	*reppBR322 Kan*^{R} *lacI* P$_{T7}$ *KdcA S*286R S287T F381H F382P L534S L535F M538T G539F P$_{T7}$ *BAPAT*	This study
p100K^{M8}B	*reppBR322 Kan*^{R} *lacI* P$_{J23100}$ *KdcA S*286R S287T F381H F382P L534S L535F M538T G539F P$_{T7}$ *BAPAT*	This study
pKH	*eppBR322 Kan*^{R} *lacI* P$_{T7}$ *KdcA* P$_{T7}$ *HIBADH*	This study
p100KY	*reppBR322 Kan*^{R} *lacI* P$_{J23100}$ *KdcA* P$_{T7}$ *ydfG*	This study
pK^{M8}H	*reppBR322 Kan*^{R} *lacI* P$_{T7}$ *KdcA S*286R S287T F381H F382P L534S L535F M538T G539F P$_{T7}$ *ydfG*	This study
p100K^{M8}H	*reppBR322 Kan*^{R} *lacI* P$_{J23100}$ *KdcA S*286R S287T F381H F382P L534S L535F M538T G539F P$_{T7}$ *HIBADH*	This study
p100K^{M8}Y	*reppBR322 Kan*^{R} *lacI* P$_{J23100}$ *KdcA S*286R S287T F381H F382P L534S L535F M538T G539F P$_{T7}$ *ydfG*	This study
p101K^{M8}H	*reppBR322 Kan*^{R} *lacI* P$_{J23101}$ *KdcA S*286R S287T F381H F382P L534S L535F M538T G539F P$_{T7}$ *ydfG*	This study

p106K^{M8}H	*reppBR322 Kan*^{R} *lacI* P$_{J23106}$ *KdcA S*286R S287T F381H F382P L534S L535F M538T G539F P$_{T7}$ *ydfG*	This study
p100K^{M8}YY	*reppBR322 Kan*^{R} *lacI* P$_{J23106}$ *KdcA S*286R S287T F381H F382P L534S L535F M538T G539F P$_{T7}$ *ydfG* P$_{T7}$ *pyc*	This study
p100K^{M8}YP	*reppBR322 Kan*^{R} *lacI* P$_{J23106}$ *KdcA S*286R S287T F381H F382P L534S L535F M538T G539F P$_{T7}$ *ydfG* P$_{T7}$ *ppc*	This study

Supplementary Table 2. Primers were used in this study.

Primers	Sequence (5′-3′)	Function
B1-F	AGAAGGAGATATACCATGAACATGCCCGAAACT GGTCCT	The construction of pB
B1-R	CTAGTCGATCAGGTTCAGGGTTTCGCCAACAGC ATC	
VB1-F	AACCTGATCGACTAGACGAAGCTTGCGGCCGCA CTCGAG	
VB1-R	GGTATATCTCCTTCTTAAAGTTAAACAAAATTAT TTC	
K1-F	AGAAGGAGATATACCATGTATACAGTAGGAGAT TAC	The construction of pK
K1-R	AAGCTTCGTCTATTTATTTTGCTCAGC	
VK1-F	AAATAGACGAAGCTTGCGGCCGC	
VK1-R	GGTATATCTCCTTCTTAAAGTTAAACAAAATTAT TTCTAG	
H1-F	AGAAGGAGATATACCATGCGTATCGCATTCATC GGCCTCGGCAAC	The construction of pH
H1-R	ATCTTTCTTGCGATAACCCTCGACGATG	
VH1-F	TATCGCAAGAAAGATCACCACCACCACCACCAC TGAGATCCGGCTG	
VH1-R	GGTATATCTCCTTCTTAAAGTTAAACAAAATTAT TTCTAG	
S286R-F	ACGGACAGATCAACAGGTGCATTCACAC	The construction of pK1 and pK9
S286R-R	TGTTGATCTGTCCGTAAGCTTCAC	
S287T-F	GACTCCACAACAGGTGCATTCACACATC	The construction of pK2
S287T-R	ACCTGTTGTGGAGTCCGTAAGCTTC	
F381H-F	ACCTCACACTTTGGAGCTTCAACAATTTTC	The construction of pK3
F381H-R	TCCAAAGTGTGAGGTTCCTTGTTCAGCAAC	
F382P-F	TCATTTCCAGGAGCTTCAACAATTTTC	The construction of pK4 and pK9
F382P-R	AGCTCCTGGAAATGAGGTTCCTTGTTC	
L534S-F	CCAAAATCACTGAAAAAAATGGGTAAATTATTT G	The construction of pK5
L534S-R	TTTCAGTGATTTTGGCGCATCTTC	
L535F-F	AAATTATTTAAAAAAATGGGTAAATTATTTGCT G	The construction of pK6
L535F-R	TTTTTTAAATAATTTTGGCGCATCTTC	
M538T-F	AAAAAAACAGGTAAATTATTTGCTGAGC	The construction of pK7
M538T-R	TTTACCTGTTTTTTTCAGTAATTTTGGCGCATC	
G539F-F	AAAATGTTTAAATTATTTGCTGAGC	The construction of pK8 and pK9
G539F-R	TAATTTAAACATTTTTTTCAGTAATTTTGGCGCA TC	
K2-F	ATGTATACAGTAGGAGATTACCTGTTAG	The construction of pKH and pK^{M8}H
K2-R	TGCAGGCGCGCCCTATTTATTTTGC	
H2-F	GAAGGAGATATACATATGCGTATCGCATTCATC GGCC	
H2-R	GTTAGCAGCCGGATCTTAATCTTTCTTGCGATAA CCCTCGACGATG	
K^{M8}-F	ATGTATACAGTAGGAGATTACCTGTTAG	The construction

K^{M8}-R	TGCAGGCGCGCCCTATTTATTTTGC	of p100K^{M8}H and p100KY
VK^{M8}-F	TAGGGCGCGCCTGCAGGTC	
VK^{M8}-R	TCCTACTGTATACATGGTATATCTCCTTATTAAA GTTAAACAAAATTATTTC	
Y1-F	GAAGGAGATATACATATGATCGTTTTAGTAACT GGAGCAAC	The construction of p100K^{M8}Y
Y1-R	GTTAGCAGCCGGATCTTACTGACGGTGGACATT CAGTC	
VY1-F	GATCCGGCTGCTAACAAAGC	
VY1-R	ATGTATATCTCCTTCTTATACTTAACTAATATAC	
PJ23101-F	CTAGCTCAGTCCTAGGTATTATGCTAGCCCTGTA GAAATAATTTTGTTTAAC	The construction of p101K^{M8}H
PJ23101-R	CTAGGACTGAGCTAGCTGTAAAATTTCCTAATG CAGGAGTCGCATAAGGGAGAG	
PJ23106-F	CTAGCTCAGTCCTAGGTATAGTGCTAGCCCTGTA GAAATAATTTTG	The construction of p106K^{M8}H
PJ23106-R	CTAGGACTGAGCTAGCCGTAAAATTTCCTAATG CAGGAGTCGCATAAGGGAGAG	
Pyc-F	AAGTATAAGAAGGAGATATACATATGAAAAAA CTACTCGTCGCCAATCGTGGAG	The construction of p100K^{M8}YY
Pyc-R	GTTAGCAGCCGGATCTTAGTCAATTTCAATCAAT AAGTCTTGTGTTTGAATG	
VPYC-F	GATCCGGCTGCTAACAAAGCCCGAAAG	
VPYC-R	CTCCTTCTTATACTTAACTAATATACTAAGATGG TTACTGACGGTGGACATTCAGTCCGGCATAGC	
PPC-F	AAGTATAAGAAGGAGATATACATATGAACGAAC AATATTCCGCATTGCGTAGTAATG	The construction of p100K^{M8}YP
PPC-R	GTTAGCAGCCGGATCTTAGCCGGTATTACGCAT ACCTGCCGCAATC	
VPPC-F	GATCCGGCTGCTAACAAAGCCCGAAAG	
VPPC-R	CTCCTTCTTATACTTAACTAATATACTAAGATGG TTACTGACGGTGGACATTCAGTCCGGCATAGC	
N20-*panD*-F	TGCGGACCTGCACTATGAGTTTTAGAGCTAGAA ATAGCAAGTTAAAATAAGGCTAG	The construction of pEcgRNA-*panD*
N20-*panD*-R	TAGTGCAGGTCCGCATGACTAGTATTATACCTA GGACTGAGCTAGCTGTC	
UP-*panD*-500-F	AGATAACCGGGATTGCCCTGCATTTG	The construction of donor dsDNA
UP-*panD*-500-R	GAGTAACCAGCCGCAGGGATAACAAAACTTCTA CCTTTCTACCCTGTCGTTAACGAAG	
Down-*panD*-500-F	TTGTTATCCCTGCGGCTGGTTACTC	
Down-*panD*-500-R	TGAGCCGCTATGCGTATCCTGTGTTG	
N20 Sequence	CATGCGGACCTGCACTATGA	Gene target

Supplementary Table 3. Synthetic promoters used in this study.

Promoters	Sequences	Relative strength [a]	Sources
BBa_J23100	ttgacggctagctcagtcctaggtacagtgctagc	1	http://parts.igem.org
BBa_J23101	tttacagctagctcagtcctaggtattatgctagc	0.70	
BBa_J23106	tttacggctagctcagtcctaggtatagtgctagc	0.47	

[a]The relative strengths of these promoters (normalized to that of BBa_J23100) were measured by Chris Anderson and the 2006 Berkeley iGEM team.

Supplementary Table 4. Summary of KdcA mutations used in this study.

Single mutation	S286R; S287T; F381H; F382P; L534S; L535F; M538T; G539F
Double mutation	S286R/S287T
Triple mutation	S286R/F382P/G539F

Quadruple mutation	S286R/S287T/F381H/F382P
Sextuple mutation	S286R/S287T/F381H/F382P/M538T/G539F
Octuple mutation	S286R/S287T/F381H/F382P/L534S/L535F/M538T/G539F

Supplementary Table 5. Decarboxylase activity of WT KdcA at varying oxaloacetate concentrations.

Oxaloacetate concentration (mM)	Initial reaction rate (μM/min)
10	0.54
20	1.28
40	3.04
50	3.4

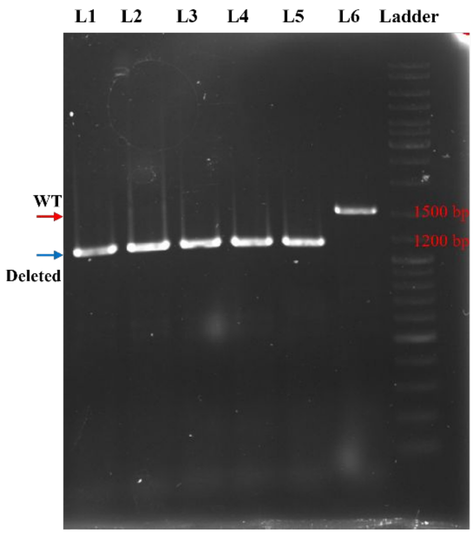

Supplementary Figure 1. Verification of *panD* gene deletion by colony PCR. Lanes L1–L5 represent PCR products from the edited colonies (Deleted) with the *panD* gene deletion, while lane L6 corresponds to the non-edited colony (WT) carrying the *panD* gene. The length of the *panD* gene is 381bp.

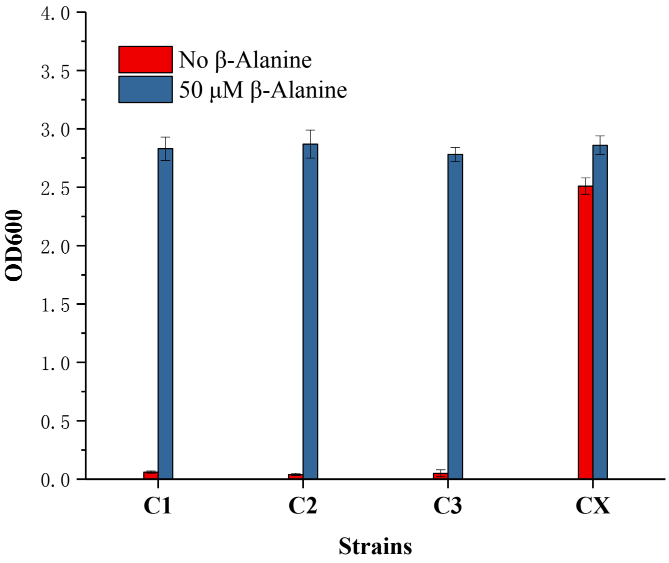

Supplementary Figure 2. The growth of *E. coli* strains was assessed with or without the complementary β-alanine biosynthetic pathway. Strains were cultivated in minimal medium for 24 hours, supplemented with or without β-alanine. C1: BL21(DE3) Δ*panD*/pBAPAT, harboring the BAPAT gene under the control of the T7 promoter; C2: BL21(DE3) Δ*panD*/pKdcA, harboring the KdcA gene under the control of the T7 promoter; C3: BL21(DE3) Δ*panD*/pKdcA-BAPAT, harboring both the KdcA and BAPAT genes under the control of the T7 promoter; CX: BL21(DE3) harboring pET28a(+).

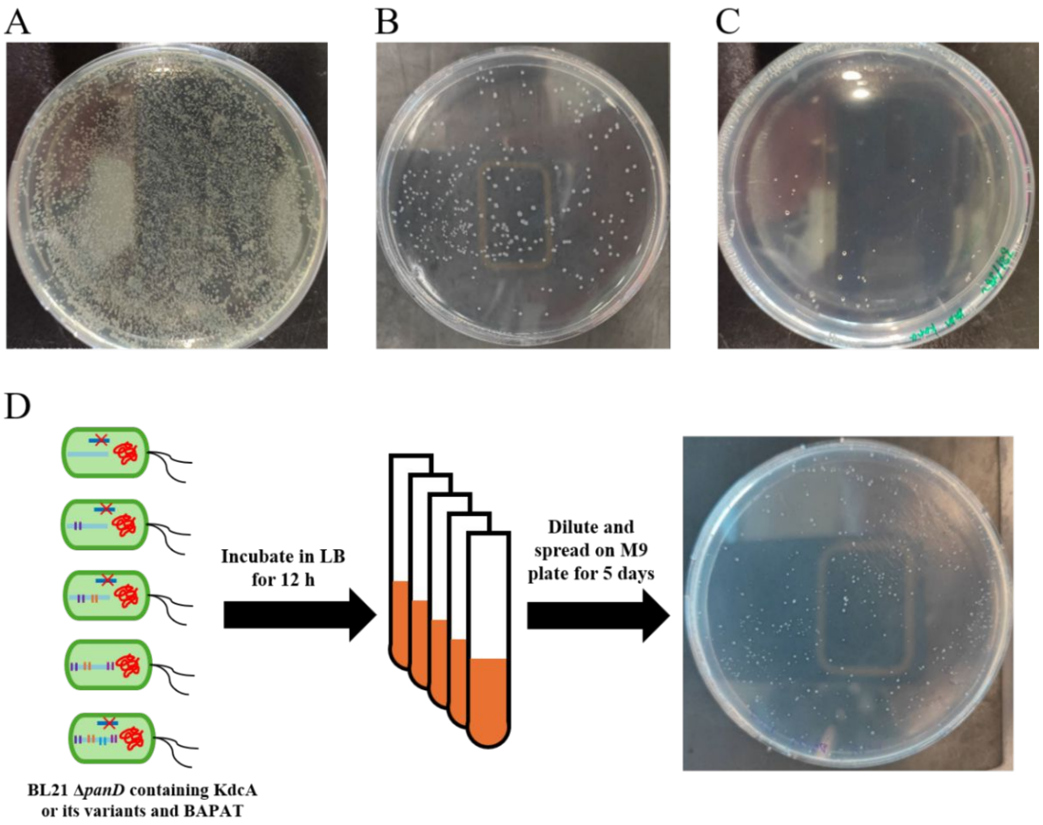

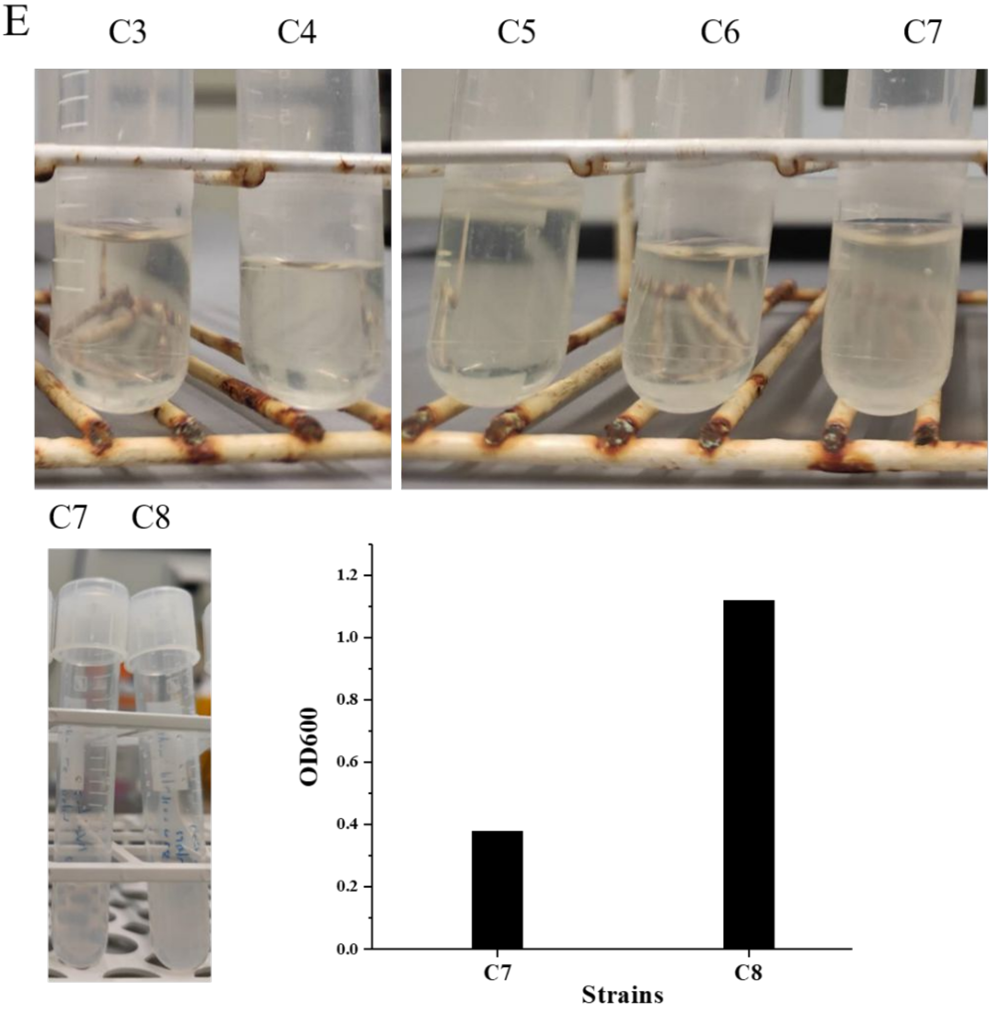

Supplementary Figure 3. The cells growth during the directed evolution of KdcA. (A) Testing the electroporation efficiency by plating a small portion (30 µL) of transformed cells on LB agar plates and incubated at 30 °C for one day; (B) Growth of *E. coli* BL21(DE3) on M9 agar plates at 30 °C for 2 days. (C) Growth of *E. coli* BL21(DE3) Δ*panD* containing the KdcA mutation library on M9 agar plates at 30 °C for 4 days. (D) Confirmatory screening of the KdcA variants identified from each round of selection. BL21(DE3) Δ*panD* strains harboring KdcA or its variants and BAPAT were cultured in LB medium for 12 hours. Then, 2 µL from each culture was mixed, serially diluted (5×10^5), and spread onto M9 agar plates, followed by incubation for five days at 30 °C. (E) Growth of engineered strains (C3–C8) in M9 liquid medium, incubated at 30 °C for 5 days. C3: BL21(DE3) Δ*panD* harboring KdcA and BAPAT under the control of the T7 promoter; C4: BL21(DE3) Δ*panD* harboring KdcA (S286R/S287T) and BAPAT under the control of the T7 promoter; C5: BL21(DE3) Δ*panD* harboring KdcA (S286R/S287T/F381H/F382P) and BAPAT under the control of the T7 promoter; C6: BL21(DE3) Δ*panD* harboring KdcA (S286R/S287T/F381H/F382P/M538T/G539F) and BAPAT under the control of the T7 promoter; C7: BL21(DE3) Δ*panD* harboring $KdcA^{M8}$ and BAPAT under the control of the T7 promoter. C8: BL21(DE3) Δ*panD* harboring $KdcA^{M8}$ and BAPAT under the control of the J23100 promoter and T7 promoter, respectively.

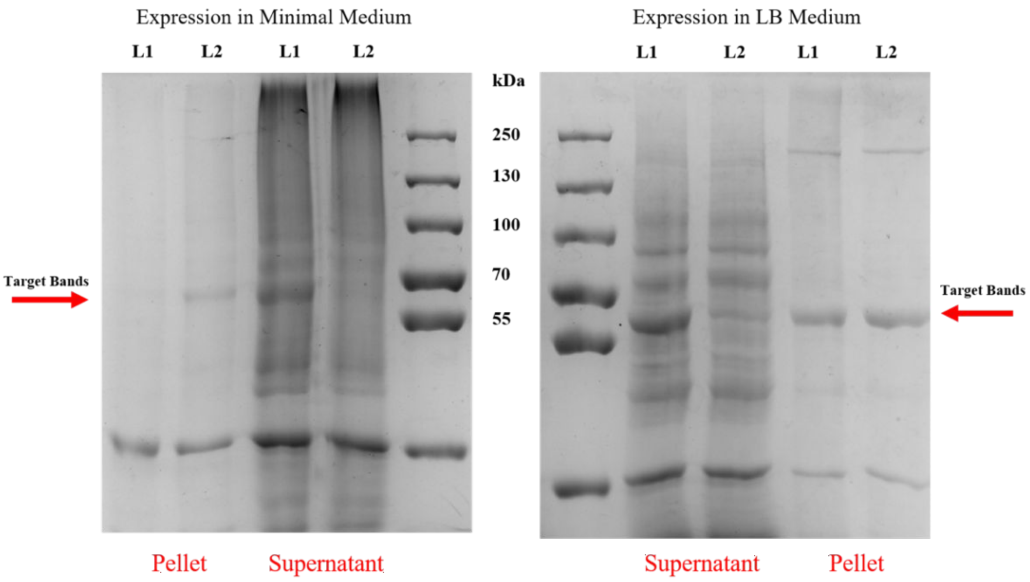

Supplementary Figure 4. Protein expression analyzed by gel electrophoresis. L1: BL21(DE3) harboring *KdcA* gene under the control of T7 promoter; L2: BL21(DE3) harboring *KdcA*^{M8} gene under the control of T7 promoter.

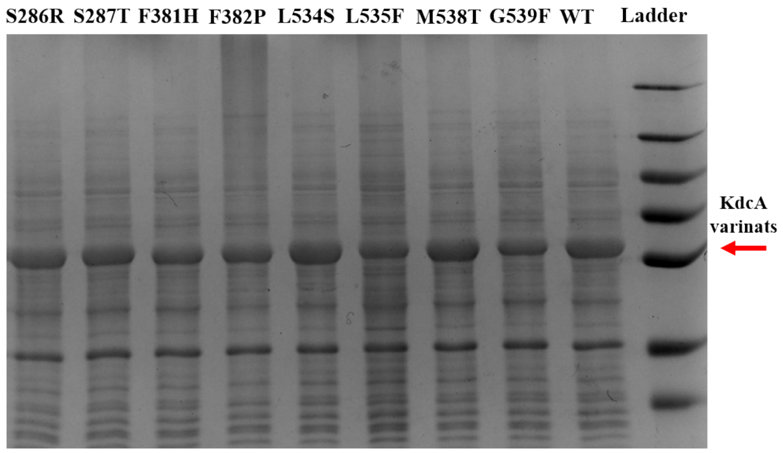

Supplementary Figure 5. Analysis of expression of KdcA variants by SDS-PAGE. Arrow marks indicate the corresponding target proteins.

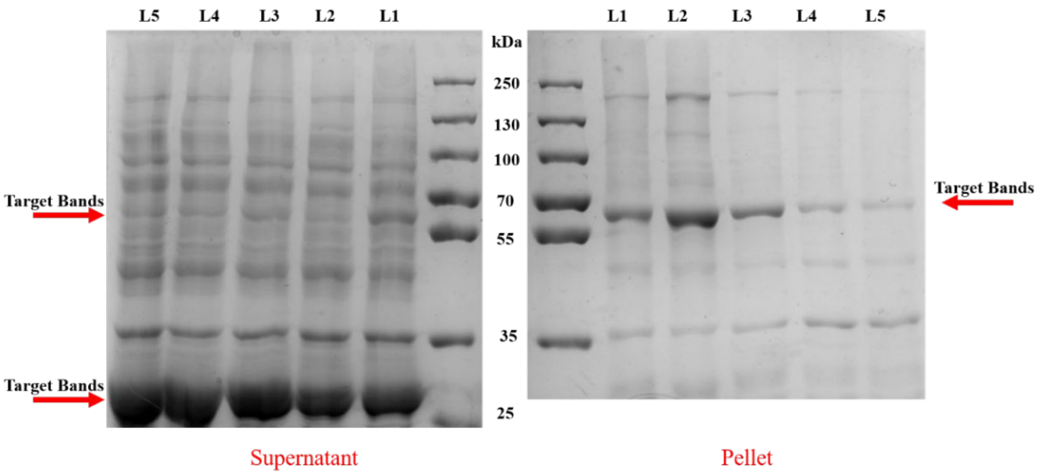

Supplementary Figure 6. Protein expression analyzed by SDS-PAGE. Cells were harvested at 24 h, diluted to OD600 = 4.0, and lysed by sonication. After centrifugation, the supernatant represents clear solution, while the pellet represents the insoluble fraction at the bottom. L1: BL21 harboring KdcA and HIBADH, the expression of KdcA was under the control of T7 promoter; L2: BL21 harboring KdcA^{M8} and HIBADH, the KdcA^{M8} expression was under the control of T7 promoter; L3: BL21 harboring KdcA^{M8} and HIBADH, the KdcA^{M8} expression is under the control of J23100; L4: BL21 harboring KdcA^{M8} and HIBADH, the KdcA^{M8} expression is under the control of J23101; L5: BL21 harboring KdcA^{M8} and HIBADH, the KdcA^{M8} expression is under the control of J23106.

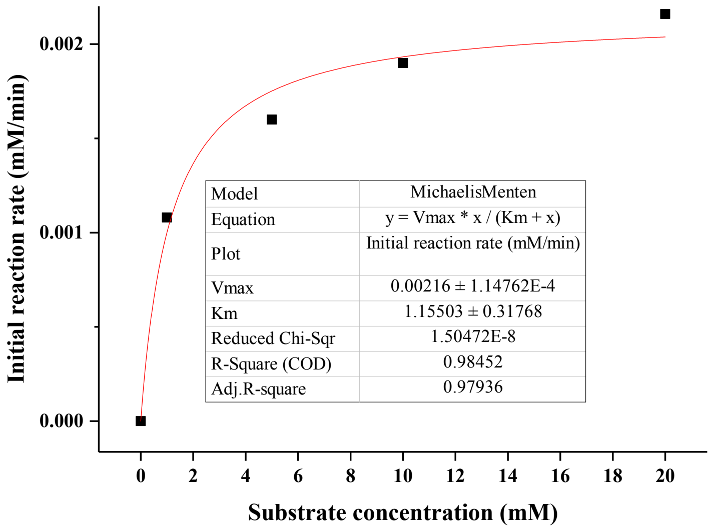

Supplementary Figure 7. Determination of K_M value of KdcA^{M8} toward oxaloacetate. Initial reaction rates were measured by monitoring the formation of 3-HP over a 60-minute period in the presence of varying concentrations of oxaloacetate and a fixed concentration of NADH. The resulting initial rates were plotted against oxaloacetate concentrations, and nonlinear regression analysis based on Michaelis-Menten kinetics was used to calculate the K_M value.

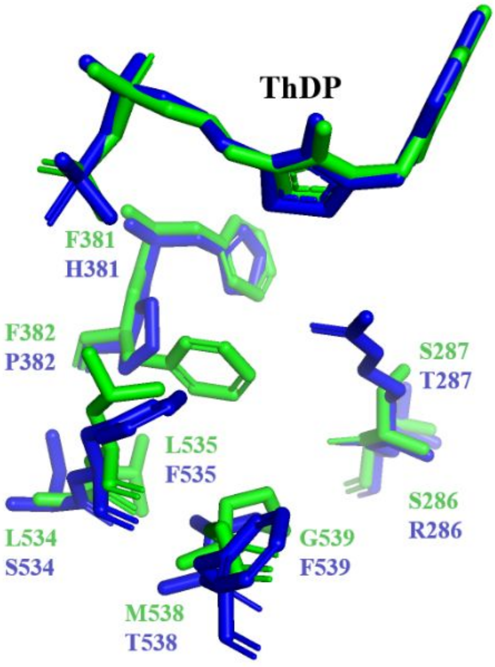

Supplementary Figure 8. Residues shaping the substrate binding pocket in WT KdcA (green) and KdcA^{M8} (blue). The structure of KdcA^{M8} was predicted with AlphaFold and aligned in PyMOL.

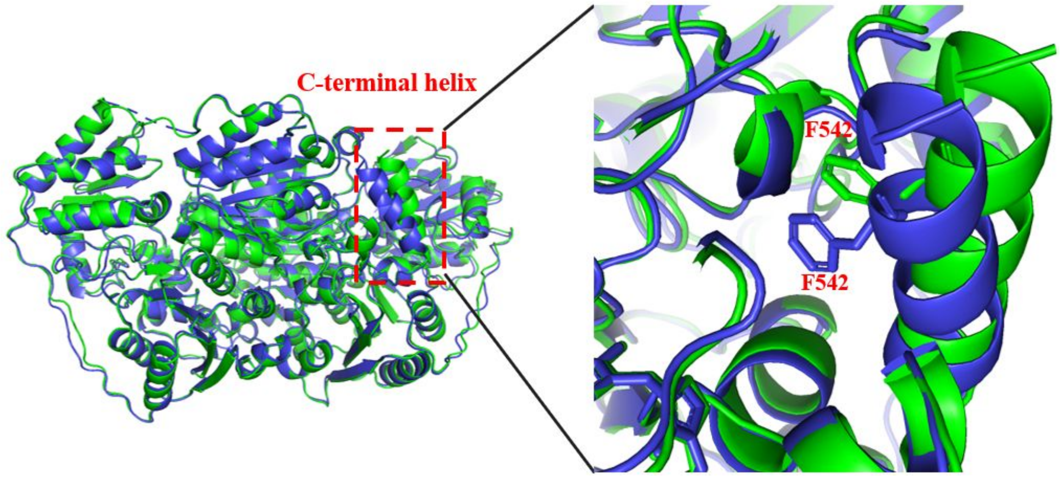

Supplementary Figure 9. The cartoon structures of KdcA^{M8} and WT KdcA are shown in green and blue, respectively. The shift of the C-terminal helix in KdcA^{M8} relative to WT KdcA is highlighted by the red rectangle.

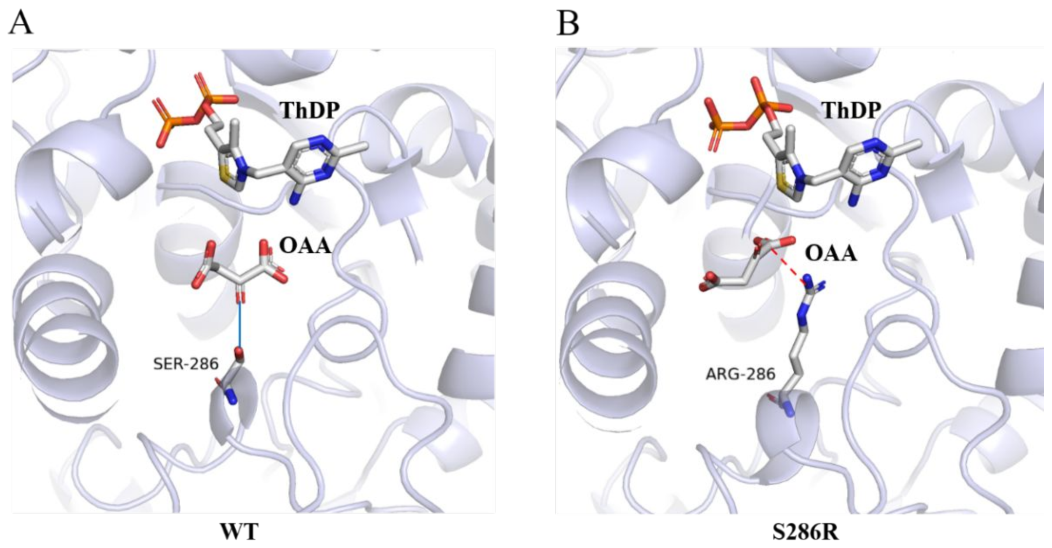

Supplementary Figure 10. Docking of oxaloacetate in the binding pocket of the structural models of WT KdcA and KdcA (S286R). Solid blue line indicates hydrogen bond; red dotted line indicates the salt bridge. AutoDock Vina was utilized for molecular docking, and the docking results were analyzed using PLIP (Protein-Ligand Interaction Profiler). ThDP: thiamine pyrophosphate; OAA: oxaloacetate.

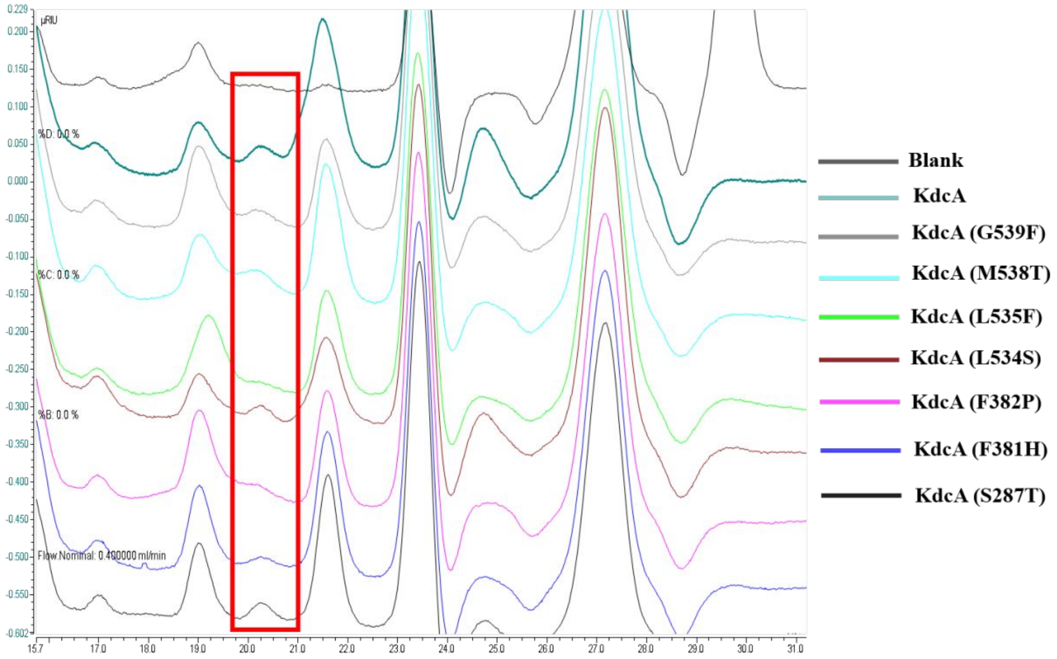

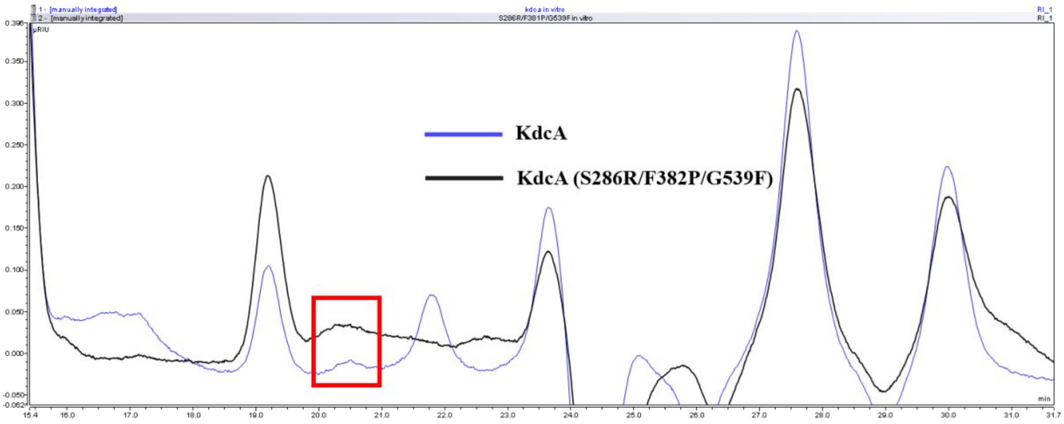

Supplementary Figure 11. Determination of 3-HP concentration by HPLC to evaluate the activity of different KdcA variants. The 3-HP peak is highlighted in a red rectangle.

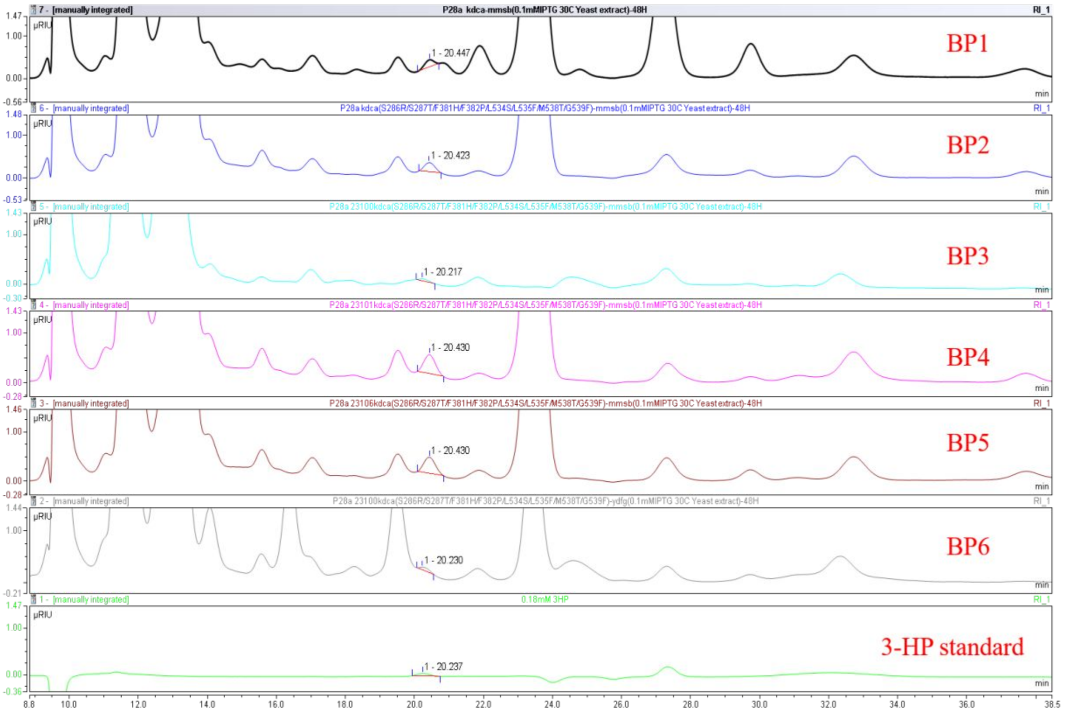

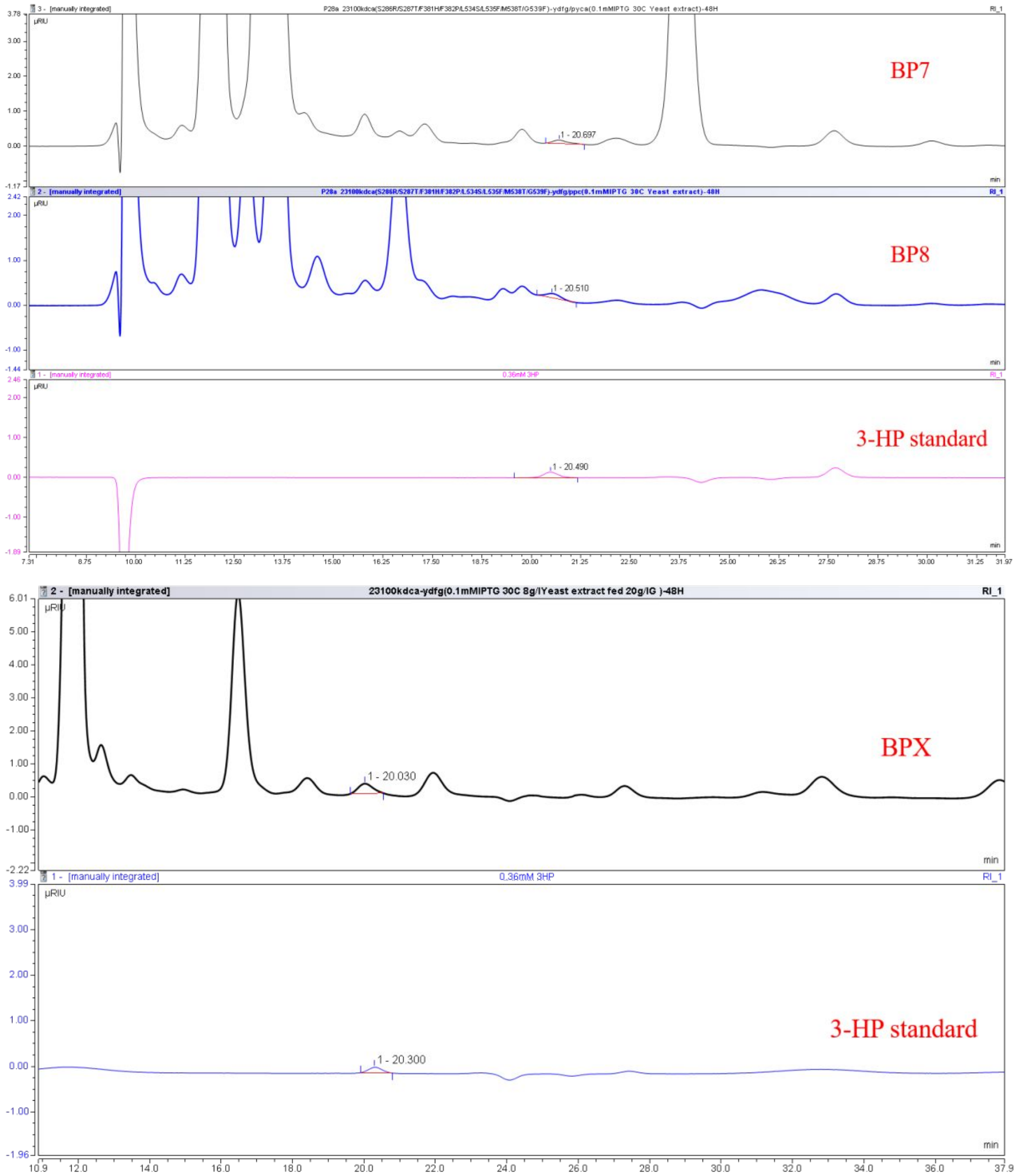

Supplementary Figure 12. HPLC analysis of 3-HP produced by strains BP1-BP8 and BPX in shake-flask cultivation at 48 hours.

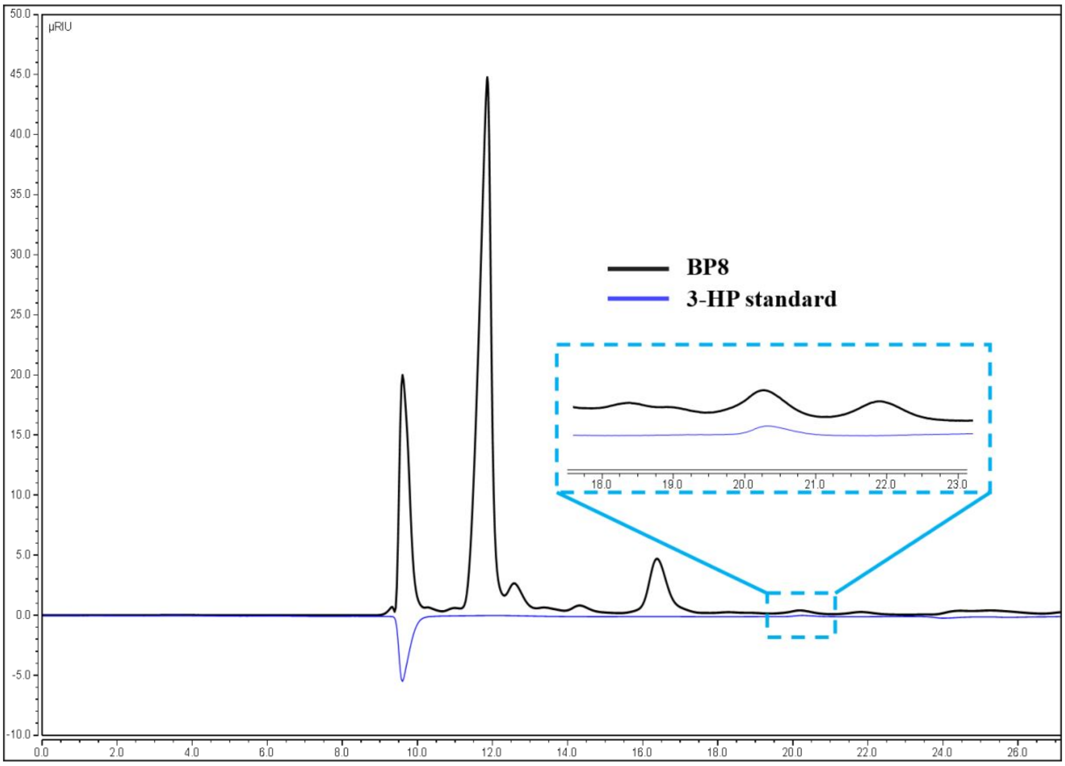

Supplementary Figure 13. HPLC analysis of 3-HP produced by strain BP8 in shake-flask cultivation at 48 hours, with peaks at a retention time of 20.18 min indicating 3-HP. HPLC analysis of 0.36 mM 3-HP (>95%), represented by blue solid lines, showing peaks at a retention time of 20.18 min corresponding to 3-HP.

DNA Sequences

KdcA* from *L. lactis

ATGTATACAGTAGGAGATTACCTGTTAGACCGATTACACGAGTTGGGAATTGAAGAAATT
TTTGGAGTTCCTGGTGACTATAACTTACAATTTTTAGATCAAATTATTTCACGCGAAGATA
TGAAATGGATTGGAAATGCTAATGAATTAAATGCTTCTTATATGGCTGATGGTTATGCTCG
TACTAAAAAAGCTGCCGCATTTCTCACCACATTTGGAGTCGGCGAATTGAGTGCGATCAAT
GGACTGGCAGGAAGTTATGCCGAAAATTTACCAGTAGTAGAAATTGTTGGTTCACCAACT
TCAAAAGTACAAAATGACGGAAAATTTGTCCATCATACACTAGCAGATGGTGATTTTAAA
CACTTTATGAAGATGCATGAACCTGTTACAGCAGCGCGGACTTTACTGACAGCAGAAAAT
GCCACATATGAAATTGACCGAGTACTTTCTCAATTACTAAAAGAAAGAAAACCAGTCTAT
ATTAACTTACCAGTCGATGTTGCTGCAGCAAAAGCAGAGAAGCCTGCATTATCTTTAGAA
AAAGAAAGCTCTACAACAAATACAACTGAACAAGTGATTTTGAGTAAGATTGAAGAAAGT
TTGAAAAATGCCCAAAAACCAGTAGTGATTGCAGGACACGAAGTAATTAGTTTTGGTTTA
GAAAAAACGGTAACTCAGTTTGTTTCAGAAACAAAACTACCGATTACGACACTAAATTTT
GGTAAAAGTGCTGTTGATGAATCTTTGCCCTCATTTTTAGGAATATATAACGGGAAACTTT
CAGAAATCAGTCTTAAAAATTTTGTGGAGTCCGCAGACTTTATCCTAATGCTTGGAGTGAA
GCTTACGGACTCCTCAACAGGTGCATTCACACATCATTTAGATGAAAATAAAATGATTTCA
CTAAACATAGATGAAGGAATAATTTTCAATAAAGTGGTAGAAGATTTTGATTTTAGAGCA
GTGGTTTCTTCTTTATCAGAATTAAAAGGAATAGAATATGAAGGACAATATATTGATAAG

CAATATGAAGAATTTATTCCATCAAGTGCTCCCTTATCACAAGACCGTCTATGGCAGGCAG
TTGAAAGTTTGACTCAAAGCAATGAAACAATCGTTGCTGAACAAGGAACCTCATTTTTGG
AGCTTCAACAATTTTCTTAAAATCAAATAGTCGTTTTATTGGACAACCTTTATGGGGTTCT
ATTGGATATACTTTTCCAGCGGCTTTAGGAAGCCAAATTGCGGATAAAGAGAGCAGACAC
CTTTTATTTATTGGTGATGGTTCACTTCAACTTACCGTACAAGAATTAGGACTATCAATCA
GAGAAAAACTCAATCCAATTTGTTTTATCATAAATAATGATGGTTATACAGTTGAAAGAG
AAATCCACGGACCTACTCAAAGTTATAACGACATTCCAATGTGGAATTACTCGAAATTAC
CAGAAACATTTGGAGCAACAGAAGATCGTGTAGTATCAAAAATTGTTAGAACAGAGAATG
AATTTGTGTCTGTCATGAAAGAAGCCCAAGCAGATGTCAATAGAATGTATTGGATAGAAC
TAGTTTTGGAAAAAGAAGATGCGCCAAAATTACTGAAAAAAATGGGTAAATTATTTGCTG
AGCAAAATAAATAG

***HIBADH* from *P. putida* KT2400**

ATGCGTATCGCATTCATCGGCCTCGGCAACATGGGCGCGCCCATGGCCCGCAACCTGATC
AAGGCCGGGCACCAACTGAACCTGTTCGACCTCAACAAGGCCGTGCTGGCCGAGCTGGCA
GAACTGGGTGGGCAGATCAGCCCGTCGCCCAAGGACGCGGCGGCCAACAGCGAGCTGGT
GATCACAATGCTGCCAGCTGCGGCCCATGTGCGCAGCGTGTACCTGAACGAGGATGGCGT
GCTGGCCGGTATTCGTGCCGGTACGCCTACCGTGGACTGCAGCACCATCGACCCCCAGAC
CGCCCGTGATGTGTCCAAGGCAGCAGCGGCCAAAGGCGTGGACCTGGGCGACGCGCCGGT
CTCCGGTGGCACTGGCGGCGCAGCGGCGGGTACCCTGACCTTCATGGTCGGTGCCAGCGC
CGAGCTGTTCGCCACGCTCAAGCCGGTACTGGAGCAGATGGGCCGCAACATCGTGCATTG
CGGTGAAGTCGGCACCGGGCAGATCGCCAAGATCTGCAACAACCTGCTGCTGGGTATTTC
GATGATTGGCGTGTCCGAGGCCATGGCCCTGGGCAACGCGCTGGGCATCGACACCAAGGT
GCTGGCCGGCATTATCAACAGCTCGACCGGGCGTTGCTGGAGTTCGGACACCTACAACCC
GTGGCCGGGCATTATCGAAACGGCACCGGCATCGCGTGGCTATACCGGCGGCTTTGGCGC
CGAACTGATGCTCAAGGATTTAGGGCTGGCCACTGAAGCGGCACGCCAGGCTCACCAACC
GGTGATCCTCGGCGCCGTGGCCCAGCAGCTGTATCAGGCCATGAGCCTGCGTGGCGAGGG
AGGCAAGGACTTCTCGGCCATCGTCGAGGGTTATCGCAAGAAAGATTGA

ydfG* from *E. coli

ATGATCGTTTTAGTAACTGGAGCAACGGCAGGTTTTGGTGAATGCATTACTCGTCGTTTTA
TTCAACAAGGGCATAAAGTTATCGCCACTGGCCGTCGCCAGGAACGGTTGCAGGAGTTAA
AAGACGAACTGGGAGATAATCTGTATATCGCCCAACTGGACGTTCGCAACCGCGCCGCTA
TTGAAGAGATGCTGGCATCGCTTCCTGCCGAGTGGTGCAATATTGATATCCTGGTAAATAA
TGCCGGCCTGGCGTTGGGCATGGAGCCTGCGCATAAAGCCAGCGTTGAAGACTGGGAAAC
GATGATTGATACCAACAACAAAGGCCTGGTATATATGACGCGCGCCGTCTTACCGGGTAT
GGTTGAACGTAATCATGGTCATATTATTAACATTGGCTCAACGGCAGGTAGCTGGCCGTAT
GCCGGTGGTAACGTTTACGGTGCGACGAAAGCGTTTGTTCGTCAGTTTAGCCTGAATCTGC
GTACGGATCTGCATGGTACGGCGGTGCGCGTCACCGACATCGAACCGGGTCTGGTGGGTG
GTACCGAGTTTTCCAATGTCCGCTTTAAAGGCGATGACGGTAAAGCAGAAAAAACCTATC
AAAATACCGTTGCATTGACGCCAGAAGATGTCAGCGAAGCCGTCTGGTGGGTGTCAACGC
TGCCTGCTCACGTCAATATCAATACCCTGGAAATGATGCCGGTTACCCAAAGCTATGCCGG
ACTGAATGTCCACCGTCAG

ppc* from *E. coli

ATGAACGAACAATATTCCGCATTGCGTAGTAATGTCAGTATGCTCGGCAAAGTGCTGGGA
GAAACCATCAAGGATGCGTTGGGAGAACACATTCTTGAACGCGTAGAAACTATCCGTAAG
TTGTCGAAATCTTCACGCGCTGGCAATGATGCTAACCGCCAGGAGTTGCTCACCACCTTAC
AAAATTTGTCGAACGACGAGCTGCTGCCCGTTGCGCGTGCGTTTAGTCAGTTCCTGAACCT
GGCCAACACCGCCGAGCAATACCACAGCATTTCGCCGAAAGGCGAAGCTGCCAGCAACCC
GGAAGTGATCGCCCGCACCCTGCGTAAACTGAAAAACCAGCCGGAACTGAGCGAAGACA
CCATCAAAAAAGCAGTGGAATCGCTGTCGCTGGAACTGGTCCTCACGGCTCACCCAACCG
AAATTACCCGTCGTACACTGATCCACAAAATGGTGGAAGTGAACGCCTGTTTAAAACAGC
TCGATAACAAAGATATCGCTGACTACGAACACAACCAGCTGATGCGTCGCCTGCGCCAGT
TGATCGCCCAGTCATGGCATACCGATGAAATCCGTAAGCTGCGTCCAAGCCCGGTAGATG
AAGCCAAATGGGGCTTTGCCGTAGTGGAAAACAGCCTGTGGCAAGGCGTACCAAATTACC
TGCGCGAACTGAACGAACAACTGGAAGAGAACCTCGGCTACAAACTGCCCGTCGAATTTG
TTCCGGTCCGTTTTACTTCGTGGATGGGCGGCGACCGCGACGGCAACCCGAACGTCACTGC
CGATATCACCCGCCACGTCCTGCTACTCAGCCGCTGGAAAGCCACCGATTTGTTCCTGAAA
GATATTCAGGTGCTGGTTTCTGAACTGTCGATGGTTGAAGCGACCCCTGAACTGCTGGCGC
TGGTTGGCGAAGAAGGTGCCGCAGAACCGTATCGCTATCTGATGAAAAACCTGCGTTCTC
GCCTGATGGCGACACAGGCATGGCTGGAAGCGCGCCTGAAAGGCGAAGAACTGCCAAAA
CCAGAAGGCCTGCTGACACAAAACGAAGAACTGTGGGAACCGCTCTACGCTTGCTACCAG
TCACTTCAGGCGTGTGGCATGGGTATTATCGCCAACGGCGATCTGCTCGACACCCTGCGCC
GCGTGAAATGTTTCGGCGTACCGCTGGTCCGTATTGATATCCGTCAGGAGAGCACGCGTC
ATACCGAAGCGCTGGGCGAGCTGACCCGCTACCTCGGTATCGGCGACTACGAAAGCTGGT
CAGAGGCCGACAAACAGGCGTTCCTGATCCGCGAACTGAACTCCAAACGTCCGCTTCTGC
CGCGCAACTGGCAACCAAGCGCCGAAACGCGCGAAGTGCTCGATACCTGCCAGGTGATTG
CCGAAGCACCGCAAGGCTCCATTGCCGCCTACGTGATCTCGATGGCGAAAACGCCGTCCG
ACGTACTGGCTGTCCACCTGCTGCTGAAAGAAGCGGGTATCGGGTTTGCGATGCCGGTTG
CTCCGCTGTTTGAAACCCTCGATGATCTGAACAACGCCAACGATGTCATGACCCAGCTGCT
CAATATTGACTGGTATCGTGGCCTGATTCAGGGCAAACAGATGGTGATGATTGGCTATTCC
GACTCAGCAAAAGATGCGGGAGTGATGGCAGCTTCCTGGGCGCAATATCAGGCACAGGAT
GCATTAATCAAAACCTGCGAAAAAGCGGGTATTGAGCTGACGTTGTTCCACGGTCGCGGC
GGTTCCATTGGTCGCGGCGGCGCACCTGCTCATGCGGCGCTGCTGTCACAACCGCCAGGA
AGCCTGAAAGGCGGCCTGCGCGTAACCGAACAGGGCGAGATGATCCGCTTTAAATATGGT
CTGCCAGAAATCACCGTCAGCAGCCTGTCGCTTTATACCGGGGCGATTCTGGAAGCCAAC
CTGCTGCCACCGCCGGAGCCGAAAGAGAGCTGGCGTCGCATTATGGATGAACTGTCAGTC
ATCTCCTGCGATGTCTACCGCGGCTACGTACGTGAAAACAAAGATTTTGTGCCTTACTTCC
GCTCCGCTACGCCGGAACAAGAACTGGGCAAACTGCCGTTGGGTTCACGTCCGGCGAAAC
GTCGCCCAACCGGCGGCGTCGAGTCACTACGCGCCATTCCGTGGATCTTCGCCTGGACGC
AAAACCGTCTGATGCTCCCCGCCTGGCTGGGTGCAGGTACGGCGCTGCAAAAAGTGGTCG
AAGACGGCAAACAGAGCGAGCTGGAGGCTATGTGCCGCGATTGGCCATTCTTCTCGACGC
GTCTCGGCATGCTGGAGATGGTCTTCGCCAAAGCAGACCTGTGGCTGGCGGAATACTATG
ACCAACGCCTGGTAGACAAAGCACTGTGGCCGTTAGGTAAAGAGTTACGCAACCTGCAAG
AAGAAGACATCAAAGTGGTGCTGGCGATTGCCAACGATTCCCATCTGATGGCCGATCTGC
CGTGGATTGCAGAGTCTATTCAGCTACGGAATATTTACACCGACCCGCTGAACGTATTGCA
GGCCGAGTTGCTGCACCGCTCCCGCCAGGCAGAAAAAGAAGGCCAGGAACCGGATCCTC

GCGTCGAACAAGCGTTAATGGTCACTATTGCCGGGATTGCGGCAGGTATGCGTAATACCG
GCTAA

pyc* from *L. lactis

ATGAAAAAACTACTCGTCGCCAATCGTGGAGAAATCGCCGTTCGTGTCTTTCGTGCCTGTA
ATGAACTCGGACTTTCTACAGTAGCCGTCTATGCAAGAGAAGATGAATATTCTGTTCATCG
CTTTAAAGCAGATGAATCTTACCTTATCGGTCAAGGTAAAAAACCAATTGATGCTTATTTG
GATATTGATGATATTATTCGTGTTGCTCTTGAATCAGGAGCAGATGCCATTCATCCAGGTT
ATGGTCTCTTATCTGAAAATCTTGAATTTGCTACAAAAGTTCGGGCAGCAGGCTTAGTTTT
TGTTGGTCCCGAGCTTCATCACTTGGATATATTTGGTGATAAAATCAAAGCAAAAGCTGCG
GCTGATGAAGCTCAAGTTCCCGGAATTCCTGGAACAAATGGTGCAGTAGATATTGACGGA
GCTCTTGAATTTGCTCAAACTTACGGATATCCAGTCATGATTAAGGCAGCATTGGGCGGCG
GCGGTCGTGGAATGCGTGTTGCGCGTAATGACGCTGAAATGCACGACGGATATGCTCGTG
CGAAATCAGAAGCTATCGGTGCCTTTGGATCTGGAGAAATCTATGTTGAAAAATACATTG
AAAATCCTAAGCATATTGAAGTTCAAATTCTTGGGGATAGTCATGGAAATATTGTCCATCT
CCACGAACGTGATTGTTCTGTTCAACGTCGAAATCAAAAAGTTATTGAAATTGCCCCAGCC
GTAGGACTCTCGCCAGAGTTCCGTAATGAAATTTGTGAAGCAGCAGTTAAACTTTGTAAA
AATGTTGGTTATGTTAATGCTGGGACGGTTGAATTTTTAGTCAAAGATGATAAGTTCTACT
TTATCGAAGTCAACCCACGTGTTCAAGTTGAACACACAATTACAGAGCTCATTACAGGTGT
AGATATTGTTCAAGCACAAATTTTGATTGCTCAAGGCAAAGATTTACATACAGAAATTGGT
CTCCCAGCACAAGCTGAAATACCACTTTTGGGCTCAGCCATTCAATGTCGAATTACTACAG
AAGACCCGCAAAATGGCTTTTTACCAGATACAGGTAAAATCGATACCTACCGTTCACCAG
GTGGTTTCGGCATTCGTTTGGACGTTGGAAATGCCTATGCTGGTTATGAAGTGACTCCCTA
TTTTGACTCGCTTTTAGTAAAAGTTTGTACCTTTGCTAATGAATTTAGCGATAGTGTACGTA
AAATGGATCGTGTGCTTCATGAATTCCGTATTCGTGGGGTGAAAACTAATATTCCATTTTT
GATTAATGTTATTGCCAATGAAAACTTTACGAGCGGACAAGCAACAACAACCTTTATTGA
CAATACTCCAAGTCTTTTCAATTTCCCACGCTTACGTGACCGTGGAACAAAGACCTTGCAC
TACTTATCAATGATTACAGTCAATGGTTTCCCAGGGATTGAAAATACAGAAAAACGCCAT
TTTGAAGAACCTCGTCAACCTCTACTTAACATTGAAAAGAAAAAGACAGCTAAAAATATC
TTAGATGAACAAGGGGCTGATGCGGTAGTTGAATATGTGAAAAATACAAAAGAAGTATTA
TTGACAGATACAACTTTACGTGATGCTCACCAGTCTCTTCTTGCCACTCGTTTGCGTTTGCA
AGATATGAAAGGAATTGCTCAAGCCATTGATCAAGGACTTCCAGAACTTTTCTCAGCTGA
AATGTGGGGTGGGGCAACCTTTGATGTCGCTTATCGTTTCTTGAATGAGTCGCCATGGTAT
CGTCTACGTAAATTACGTAAACTCATGCCAAATACCATGTTCCAAATGCTTTTCCGTGGTT
CAAATGCAGTTGGATATCAAAACTATCCTGATAATGTTATTGAAGAATTTATCCGTGTAGC
TGCACATGAAGGAATCGATGTCTTCCGTATCTTTGATAGCCTCAACTGGTTACCACAAATG
GAAAAATCAATCCAAGCAGTGCGTGACAATGGAAAAATTTCCGAAGCAACCATTTGTTAT
ACAGGAGATATCCTTGACCCAAGTCGACCAAAATATAATATCCAATACTACAAAGATTTG
GCAAAAGAATTAGAAGCTACTGGGGCTCATATACTTGCCGTTAAAGATATGGCGGGCTTG
TTGAAACCTCAAGCGGCATATCGCTTGATTTCTGAATTAAAAGATACAGTTGACTTACCAA
TTCACTTGCATACACATGATACTTCAGGAAATGGTATTATTACCTATTCTGGTGCAACTCA
AGCAGGAGTAGATATTATTGATGTGGCAACGGCCAGTCTTGCTGGTGGAACTTCTCAACCT
TCAATGCAATCAATTTATTATGCCCTTGAACATGGTCCCCGTCATGCTTCAATTAATGTGA
AAAATGCAGAGCAAATTGACCACTATTGGGAAGATGTGCGTAAATATTATGCACCTTTTG

AGGCAGGAATTACGAGCCCACAAACGGAAGTTTACATGCATGAGATGCCTGGCGGACAGT
ATACTAACTTGAAATCTCAAGCAGCAGCTGTTGGACTTGGACATCGTTTTGATGAAATCAA
ACAAATGTATCGTAAAGTAAACATGATGTTTGGCGATATCATTAAAGTAACTCCTTCATCA
AAAGTAGTTGGTGATATGGCACTCTTTATGATTCAAAACGAATTGACAGAAGAAGATGTC
TATGCGCGAGGAAATGAGCTCAACTTCCCTGAATCAGTAGTTTCATTCTTCCGTGGTGATT
TAGGTCAGCCTGTTGGCGGCTTTCCAGAAGAATTACAAAAAATTATTGTAAAAGACAAAT
CGGTCATTACGGATCGTCCAGGATTACATGCCGAAAAAGTTGATTTTGCAACTGTAAAAG
CTGACTTGGAACAAAAAATTGGTTATGAACCAGGTGATCATGAAGTTATCTCTTATATTAT
GTATCCACAAGTTTTCCTTGATTATCAAAAAATGCAAAGAGAATTTGGAGCTGTCACACTA
CTTGATACGCCAACTTTCTTACACGGAATGCGTCTCAACGAAAAAATTGAAGTCCAAATTG
AAAAAGGTAAAACGCTCAGCATTCGTTTAGATGAAATAGGAGAACCTGACCTCGCTGGAA
ATCGTGTGCTCTTCTTTAACTTGAACGGTCAGCGTCGTGAAGTTGTGATTAATGACCAATC
CGTTCAAACTCAGATTGTAGCTAAACGTAAGGCCGAAACAGGTAATCCAAACCAAATTGG
AGCAACTATGCCCGGTTCTGTTCTTGAAATCCTAGTTAAAGCTGGAGATAAAGTTAAAAA
AGGACAAGCTTTGATGGTTACTGAAGCCATGAAGATGGAAACGACCATTGAGTCACCATT
CGATGGAGAAGTTATTGCCCTTCATGTTGTCAAAGGTGAAGCCATTCAAACACAAGACTT
ATTGATTGAAATTGACTAA
